# Young‐Of‐Year Atlantic Cod and Saithe Differ in Individuality and Structure of Movement Traits

**DOI:** 10.1002/ece3.74149

**Published:** 2026-08-02

**Authors:** Guðbjörg Ásta Ólafsdóttir, Michelle L. Valliant

**Affiliations:** ^1^ University of Iceland Research Centre of the Westfjords Bolungarvik Iceland

**Keywords:** behavioral plasticity, individuality, juvenile fish, locomotion, social context

## Abstract

Individual differences in behavior impact ecological interactions and potentially influence life‐history strategies. However, comparative studies examining how behavioral variation is structured in closely related, ecologically similar species remain limited. We investigated the expression of movement traits and the structure of behavioral variation in young‐of‐year (YOY, 0‐group) Atlantic cod ( *Gadus morhua* ) and saithe ( *Pollachius virens* ) using repeated laboratory trials over 3 months in solitary open‐field and mirror contexts, followed by additional paired conspecific trials using the same experimental contexts. Several movement traits were quantified, and activity and acceleration were analyzed using Bayesian mixed‐effects models to estimate species differences, context‐dependent plasticity, and among‐individual variation across time and social contexts. Species differed modestly in behavioral expression; saithe were quicker to explore and exhibited higher acceleration than Atlantic cod. Cod activity increased, and acceleration declined over time, whereas saithe exhibited little change. Context produced contrasting effects for both species: mirror exposure reduced activity (no change in acceleration), paired trials increased activity in both species, while acceleration showed no clear change in cod but decreased in saithe. Beyond species mean responses, species differed in the organization of behavioral variation. Cod exhibited stronger and more structured among‐individual differentiation, including strong intercept–slope relationships, but weaker coupling among movement traits. Saithe showed tighter integration among movement traits, higher residual variability, and more variable responses across contexts. These results demonstrate that closely related, ecologically similar gadoids differ not only in behavioral expression but in how behavioral variation is partitioned among and within individuals, and this is apparent even during the first months of life. The noted differences align with ecological contrasts between demersal, facultatively social Atlantic cod and pelagic, more strongly shoaling saithe.

## Graphical abstract

We examined movement behavior in young‐of‐year Atlantic cod and saithe using repeated laboratory trials across ontogeny and social contexts. While mean behavioral differences were modest, the species diverged strongly in how behavioral variation was structured: cod showed greater among‐individual differentiation and context‐dependent plasticity, whereas saithe exhibited tighter coupling among movement traits and higher residual variability.

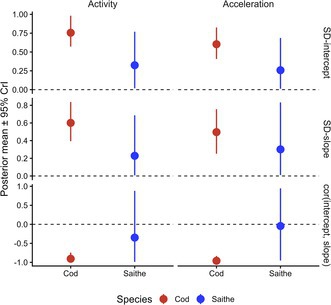

## 1 Introduction

Individual differences in behavior play a significant role in shaping ecological interactions, for example, through foraging and dispersal (Clobert et al. 2009; Chapman et al. 2011), influencing individual fitness (Dingemanse and Réale 2005) and ultimately population‐level processes. Behavior often constitutes a first response to the environment, and behavioral plasticity can allow individuals to adjust appropriately to variable conditions (Snell‐Rood 2013; Dingemanse and Wolf 2013). Therefore, many animals show context‐dependent behavior, and in heterogeneous or unpredictable environments, behavioral plasticity can be adaptive (Dingemanse and Wolf 2013). At the same time, animal populations are often composed of individuals that differ consistently in behavioral traits, commonly referred to as animal personality (Dingemanse and Réale 2005). Individuality structures population variation in behavior from within individuals to among individuals. Moreover, behavioral traits are often correlated at the individual level (referred to as behavioral syndromes), potentially constraining the combinations of behaviors that an individual expresses (Sih et al. 2004; Dochtermann and Dingemanse 2013). Together, these processes highlight a fundamental structural tension between behavioral flexibility and behavioral constraint.

Empirical evidence of individuality in behavior has grown rapidly in the past decades, but most studies are still based on model organisms (Rosenthal et al. 2017) and on adult stages, whereas a focus on early life stages can reveal novel insight on the onset and permanency of individuality (Roy and Arlinghaus 2022; Scherer et al. 2023; Dellinger et al. 2023). Atlantic cod ( *Gadus morhua* ) and saithe ( *Pollachius virens* ) provide a relevant comparative system in this context. While among‐individual variation of behavior has been demonstrated for both species, for example, for Atlantic cod in early exploration (Beukeboom et al. 2022) and homing behavior (Thorsteinsson et al. 2012), and for saithe in diel movement and activity (Skilbrei and Otterå 2016), the species life‐history strategies make it intuitively appealing to compare their behavioral structure. Saithe are strong schoolers that undertake long‐range movements (Homrum et al. 2012, 2013; Myksvoll et al. 2021), and forage primarily in the pelagic zone, or as early juveniles through transient use of epibenthic coastal waters (Rangeley and Kramer 1995). Conversely, Atlantic cod are demersal, facultatively social, and exhibit pronounced heterogeneity in migratory tendency and residency strategies (Robichaud and Rose 2004). Behavioral heterogeneity may be a critical part of Atlantic cod species resilience, allowing individuals to exploit a wide range of habitats and geographic areas (Robichaud and Rose 2004; Knutsen et al. 2018). Juvenile saithe and Atlantic cod occur in sympatry in nursery habitats around Iceland and use broadly similar habitats and resources during the first two or three years of life, yet differ markedly in their movement around these habitats, with cod showing facultative, condition‐dependent persistence while juvenile saithe occur in more transient shoals (Ólafsdóttir and Nickel 2026).

Behavioral variation may be particularly important during the early‐life stages of fish when mortality is highest. Moreover, the first year of life is a period of substantial change, involving shifts in habitat, depth distribution, and predator exposure that require rapid adjustment of movement, foraging, and anti‐predator behavior. There is some evidence that specific strategies are consistent across life stages, such as young‐of‐year (YOY) resident Atlantic cod using shallower waters and being bolder than the same age migratory cod ecotypes (Ólafsdóttir et al. 2023; Beukeboom et al. 2023), but it is generally supported that behavioral plasticity is greatest in early life (West‐Eberhard 1989; Nettle and Bateson 2015; Polverino et al. 2016). Underlying behavioral structure can be critical in the competitive, high‐risk environment experienced by juvenile fish, as decoupling among behavioral traits and behavioral plasticity may enable flexible, facultative responses to local conditions. Conversely, more rigid expressions and strong coupling of movement‐related behaviors may also be advantageous, for example, by facilitating coordinated escape responses.

Specifically, among‐individual variation in movement traits may underlie both base behavioral expression and ecological consequences. Fish movement, or swimming, reflects distinct modes: sustained cruising and burst or fast‐start performance, which differ in both kinematic and energetic characteristics (Weihs 1973; Webb 1984). Sustained swimming involves energetic, efficient, steady propulsion and supports routine activities such as exploration or shoaling over extended periods (Weihs 1973; Domenici and Blake 1997). In contrast, burst swimming is characterized by rapid acceleration and deceleration and is energetically costly, but important for escape responses, prey capture, and fine‐scale maneuvering (Domenici and Blake 1997). Acceleration is therefore a key part of swimming mechanics (Blake 2004). Strategies that reduce energetic costs associated with swimming may differ among species depending on their life history, including tactics such as resting or sheltering in structurally complex habitats or coordinated movement within cohesive shoals (Di Santo and Goerig 2025), and swimming shows substantial interspecific variation, with pelagic species showing higher activity levels than benthic species (Videler 1993; Webb et al. 1994; Kolok et al. 1998). Therefore, differences in traits that reflect swimming mode provide a useful framework for examining behavioral strategies and behavioral structure across fish species.

Controlled laboratory experiments remain necessary for resolving the intrinsic structure of behavioral variation. Laboratory environments simplify conditions, allowing intrinsic behavioral variation to be quantified more independently of environmental variation. Such experiments therefore complement field studies by identifying behavioral mechanisms that may generate ecological and evolutionary consequences in the field. In the current study, we used controlled laboratory trials to examine a suite of movement traits in YOY Atlantic cod and saithe, over a 3‐month period, and across distinct contexts. Using repeated measures of individually identifiable fish exposed to solitary open‐field, solitary mirror exposure, and live conspecific conditions, we quantify species‐specific patterns of trait expression and context‐dependent variation, as well as among‐individual variation and individual plasticity. Specifically, we examine whether individuality, behavioral plasticity, trait coupling, and within‐individual variation differ between the two species. In nature, Atlantic cod exhibits heterogeneous life‐history and movement strategies, whereas saithe are characterized by stronger shoaling behavior and more uniform population‐level movement patterns. We therefore predicted that juvenile Atlantic cod would (1) exhibit greater among‐individual variation and (2) greater behavioral plasticity across contexts, whereas juvenile saithe would (3) exhibit tighter coupling among movement traits.

## 2 Materials and Methods

### 2.1 Behavioral Experiments

The YOY gadoids, cod (45) and saithe (16) used in the experiments were collected using a beach seine (length = 10 m, depth = 1.5 m, mesh = 6 mm) in October 2020 and September 2021 in Seyðisfjörður, Westfjords of Iceland (65.995094, −22.940905), except five cod caught as by‐catch during the Marine and Freshwater Research Institute (MFRI) annual shrimp trawling survey in October 2021 within Ísafjarðardjúp (66.083333, −22.800000). At capture, the standard length of cod was on average 5.82 cm, range = 4.36–9.97, and the standard length of saithe was on average 7.41 cm, range = 6.27–9.14. The juveniles were transported to a laboratory in Bolungarvik, Iceland (66.156631, −23.249552), and housed individually in 9.5‐l tanks (∼approximately 29 × 21 × 19 cm, water level 16 cm, Aquaneering Inc.). Individual housing ensured known identity throughout repeated trials and prevented prior interactions among individuals before testing. However, the holding conditions included olfactory and visual exposure to the other fish in the system, but this was species specific, that is, cod could see other cod and saithe could see other saithe. The recirculating system contained freshwater mixed with marine salt to achieve a natural salinity of 32‰ ± 1‰, a temperature of 10°C ± 0.5°C, ammonia levels of < 0.5 ppm, oxygen levels of 10.4 ± 0.1 mg/L, and a constant photoperiod of 12:12 (7 AM‐7 PM GMT). The water circulated through the system, passing through all the tanks, a biofilter, sieves (mesh size 25 μm), and a UV light for sterilization. Each individual tank included a gray PVC pipe (knee) to provide shelter for the fish. Fish were fed on an alternating schedule of shrimp, supplemented with cod liver oil, and defrosted bloodworms, with fasting on every third day. This feeding regime promoted natural growth rates while reducing the risk of overfeeding, which could affect swim bladder function and, consequently, swimming performance. On feeding days, fish were fed *ad libitum*. The standard length and weight of the experimental juveniles were measured monthly to ensure that they followed natural growth trajectories. At the end of the experiments, the juveniles were euthanized by overdose of phenoxyethanol.

### 2.2 Behavioral Tests

**FIGURE 1:**
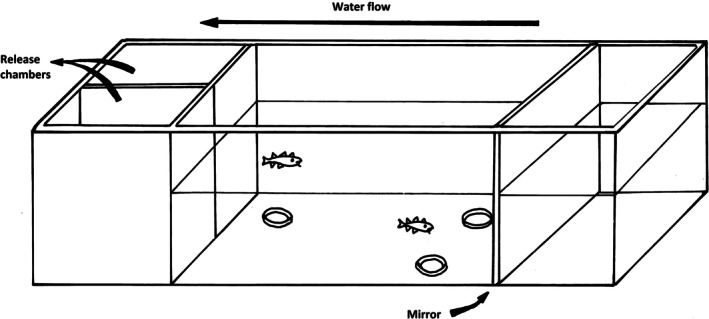
Experimental arena showing the context used in the laboratory trials. Open‐field trials included the three petri dishes shown, the mirror trials a mirror on the far wall of the arena. Either a single or two paired fish were tested in each trial. The tank was 240 × 40 cm with the arena being 160 × 40 cm.

The core dataset consists of repeated individually identifiable trials conducted once a month across 3 months. Note that in 2020 the first trial was on October 19th, but in 2021, the first trial was on September 15th. A total of 45 cod and 16 saithe were tested. Fish were gently netted from their holding tank and placed into a shelter compartment within the arena (40 × 40 × 40 cm; water depth 16 cm). After 5 min of acclimation, the shelter door was lifted. Latency to exit the shelter was recorded (maximum 300 s). If fish did not exit voluntarily within 5 min, they were gently guided into the arena and assigned the maximum latency score. The open arena was visually isolated on all sides with a plastic screen covering the exterior surface to prevent outside visibility during the experiments. The open area had two petri dishes with bloodworms attached (3 worms each). One petri dish was near the door of the shelter compartment, and the other was at the opposite end of the tank (Figure 1). Once in the open arena, the shelter door was closed, and the fish was allowed to move freely for 10 min (open‐field test). The mirror test was initiated in the same way as the open‐field test, but included a mirror placed along one short wall of the arena, opposite the shelter entrance, and no petri dishes with bloodworms were used to avoid confounding responses to the mirror stimulus. Fish were also exposed to the mirror test for 10 min. A mirror provided a standardized visual stimulus commonly used in behavioral ecology and often interpreted as a social cue (Beukeboom et al. 2023). However, responses to a mirror image may also reflect vigilance, responses to an unfamiliar moving stimulus, or other behavioral processes. Latency to explore was extracted at the onset of both tests.

We additionally conducted paired trials with live conspecifics, allowing comparison between responses to a standardized visual stimulus and a more natural dynamic social interaction. The paired tests involved matching two individuals of the same species based on small but notable body size differences (roughly 1.0 cm standard length difference), allowing consistent identification both by eye and in automated data extraction. For this reason, (1) only a subset of the fish was tested, (2) the pair identity was the same in all trials, and (3) we first paired the fish at the end of the 3‐month trial. Before commencing with the paired trials, the pairs were moved to a joint holding tank of 70 L (∼174 × 21 × 19 cm, water level 16 cm). Feeding and fish maintenance otherwise remained the same as described above. At the onset of the trials the fish were placed into two partitioned compartments of the shelter. This facilitated body‐size separation and precise identification of which individual entered the open arena first, based on its compartment of origin. The paired trials again consisted of three repeated trials, but they were executed in succession over 4–7 days. The trials were otherwise identical to the tests of solitary fish; that is, two similarly sized individuals were introduced simultaneously into the release chamber to measure latency to explore, followed by either the open‐field test or the mirror test. A total of 18 cod (9 pairs) and 14 saithe (7 pairs) were included in paired trials.

### 2.3 Behavioral Metrics Extracted From Video Recordings

Behavior of individually identifiable fish was quantified from overhead video recordings of laboratory trials using automated tracking software (LoliTrack v5.10.0). For each trial, fish position was tracked continuously, and a suite of movement and exploration related metrics was extracted. Latency to explore (LE) was time (sec) to exiting the shelter. If the fish did not leave the score was set to 300 s. The compartment was divided into four equal squares and the visits to each counted (movement across squares). Mean acceleration was calculated as the average absolute acceleration over the trial, swimming speed was quantified as the mean speed across the trial, active time was the total time spent moving, and the distance traveled was the total path length. These metrics reflect complementary components of movement within the experimental arena.

### 2.4 Ethics Note

The number of fish and the procedures used (fishing, handling and behavioral tests) adhered to strict ethics guidelines, but ethics committee approval for the research project (behavioral observation without physical intervention) was not required by Icelandic regulation (Act No. 55/2013 on Animal Welfare).

### 2.5 Statistical Analysis

**FIGURE 2:**
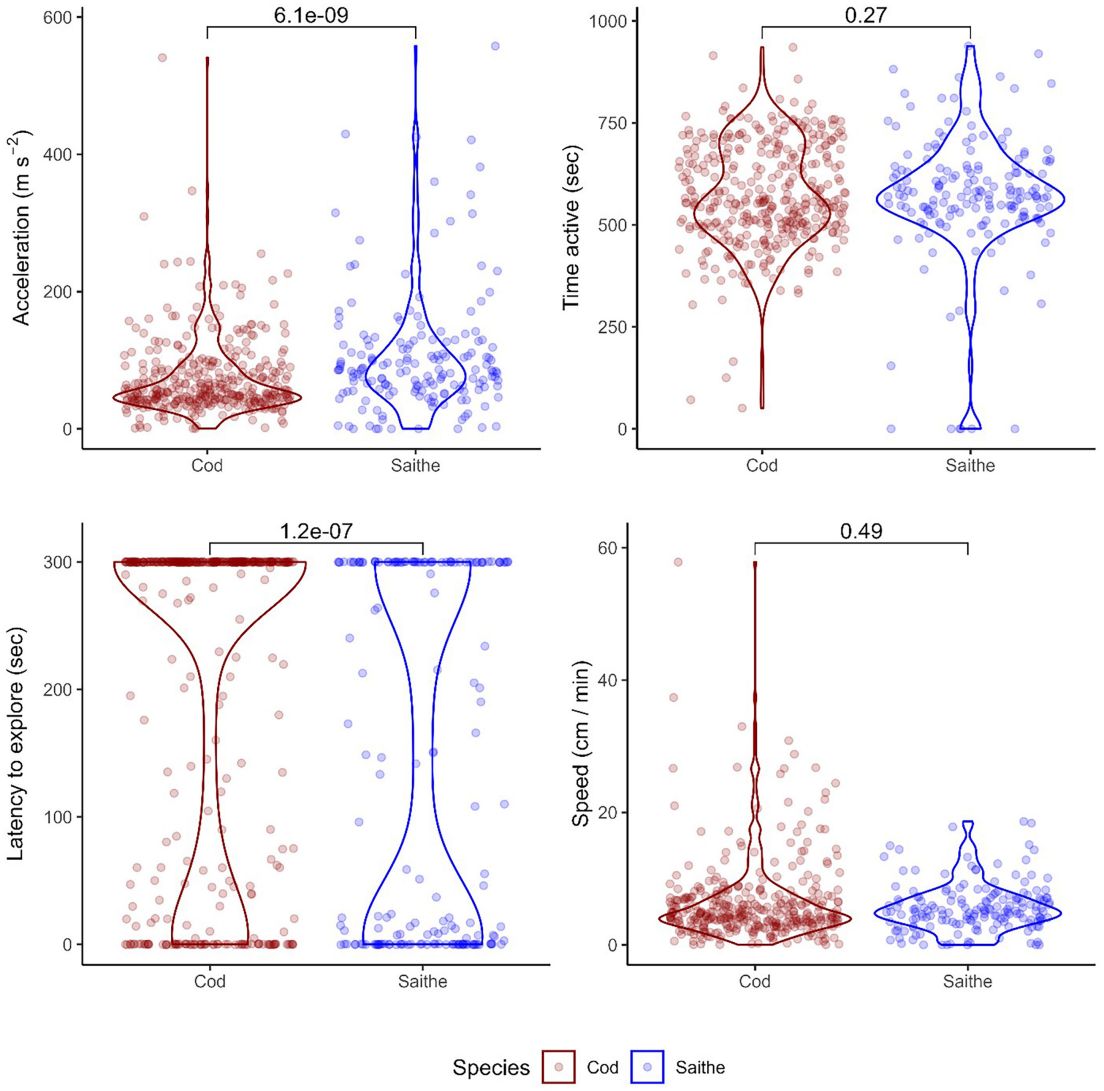
Species differences in latency to explore, activity, acceleration, and speed across all trials. Values represent individual trial observations. Boxes show median and interquartile range; whiskers show range excluding outliers.

**FIGURE 3:**
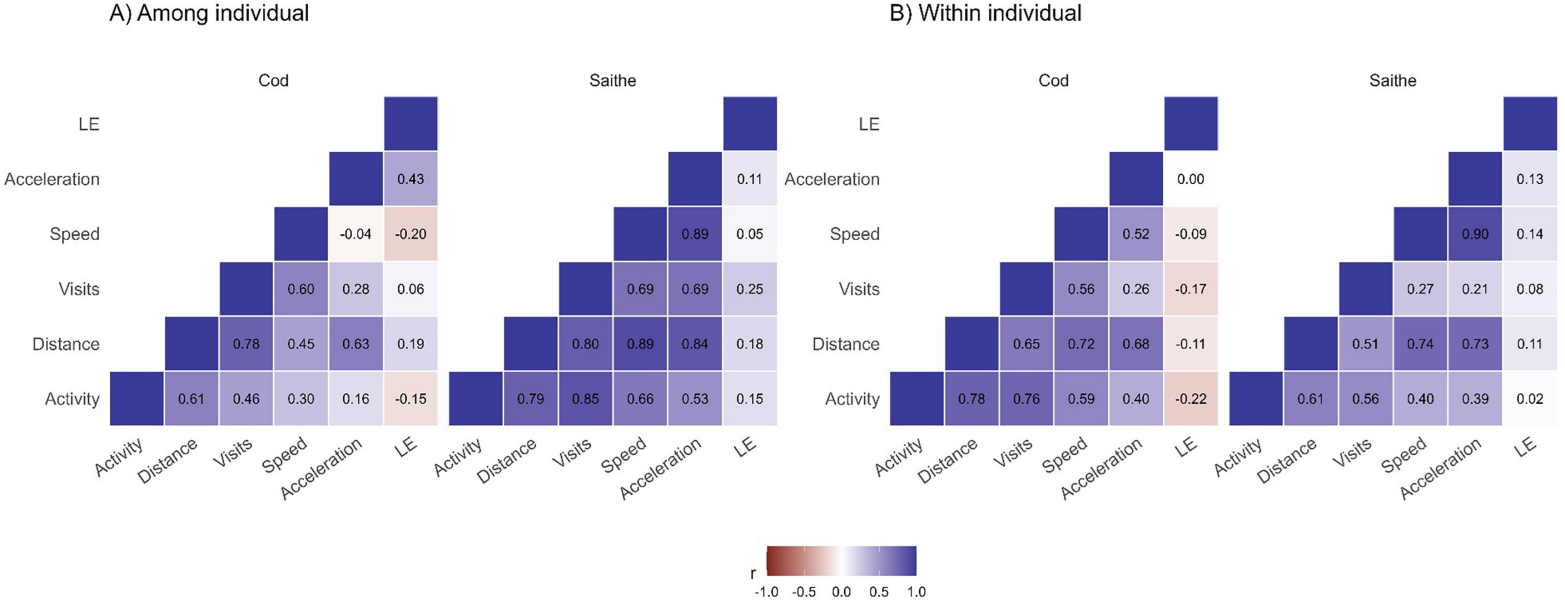
Among‐ and within‐individual correlations among movement traits in juvenile Atlantic cod and saithe. Panel A shows correlations based on individual mean trait values; panel B shows within‐individual correlations after accounting for repeated observations. Saithe showed tighter clustering among movement traits, especially involving speed and acceleration, whereas cod exhibited weaker or less consistent coupling.

Data wrangling and visualization were conducted in R (R Core Team 2025) using the *tidyverse* (Wickham et al. 2019), *posterior* (Bürkner et al. 2025), and *patchwork* (Pedersen 2020). Initial data exploration included visualizing the distribution of movement traits at the species level (Figure 2), testing for species differences using Kruskal–Wallis tests, and visualizing trait correlations (Figure 3). We visualized correlations among behavioral metrics using correlation heatmaps. First, to describe among‐individual trait coupling, repeated observations were averaged within individuals, and Spearman correlation matrices were calculated from these mean trait values. Second, each observation was centered by subtracting the individual mean for that trait, and Spearman correlation matrices were calculated from these centered values. Correlations were calculated using complete observations for activity, distance, number of arena visits, swimming speed, acceleration, and latency to explore. These heatmaps were used as descriptive summaries of trait organization and to guide trait selection for model analysis.

Based on the exploratory analysis, we focused subsequent model‐based analyses on standardized activity, measured as z‐transformed active time and speed‐corrected acceleration. Although raw acceleration was not strongly correlated with general activity, it was positively associated with swimming speed. Importantly, this speed–acceleration correlation differed between species, being strong in saithe but weak in cod. We used speed‐corrected acceleration in analysis to avoid interpreting species differences in swimming speed as differences in burst‐like movement. Speed‐corrected acceleration was calculated as the residuals from a linear regression of average acceleration on mean speed at the trial level. Residual acceleration reflects acceleration beyond that expected for the observed swimming speed. The values were scaled (z‐transformed) within the dataset being used for each model (paired, solitary).

### 2.6 Statistical Modeling

The statistical modeling (all three models described below) was performed using the Bayesian framework implemented in the R package *brms* (Bürkner 2017). All models were fitted using Hamiltonian Monte Carlo sampling (4 chains, 6000 iterations per chain, 3000 warm‐up), yielding 12,000 posterior draws. We used default priors in all models. Convergence was assessed using R̂ statistics and effective sample sizes. Posterior means and 95% credible intervals (CI) are reported. We considered directional effects to be well supported when the 95% CI did not overlap zero.

In the mixed‐effects models, fixed effects describe average trait expression and population‐level (species) changes across time or context, or effects of length, growth rate, and condition. Individual‐level random intercepts estimate among‐individual differences in behavior, which we also refer to as individuality. Individual‐level random slopes estimate among‐individual differences in temporal or context‐dependent change, which we also refer to as individual variation in plasticity or responsiveness. Intercept‐slope correlations indicate whether the baseline behavioral type predicts responsiveness and are interpreted as structured individuality, structured plasticity or structured reaction norms. In the multivariate across time models, residual correlations between activity and acceleration were interpreted as within‐individual trait coupling. Residual variation describes the within‐individual variability in behavior remaining after accounting for fixed effects and individual‐level intercepts and slopes.

First, we estimated raw repeatability, measured as the intraclass correlation coefficient (ICC), for pooled observations of activity and acceleration as a descriptive measure of behavioral consistency without context. This model included no context or fixed effects.

#### 2.6.1 State Dependent Variation in Movement Traits Across Time (Model 1)

To examine changes in the movement traits across time, as well as their association with fish state (length, Fulton's condition factor—K, and growth rate), we fitted a multivariate Bayesian mixed‐effects model for Atlantic cod and saithe separately. Species were analyzed independently due to differences in size distributions. For each species, acceleration and activity were modeled jointly. Both response variables followed a Gaussian error distribution after standardization. Modeling the traits jointly allowed estimation of the residual correlation between acceleration and activity, but model estimation of among‐individual correlation was not attempted due to the low sample size of saithe. Fixed effects included trial number (monthly trials centered), individual length at trial, growth rate across the 3 months, and Fulton's condition factor (K) at trial. Individual identity (ID) was included as a random intercept, and random slopes across trial number were fitted for all individuals.

#### 2.6.2 Transition From the Open‐Field to Mirror Test (Model 2)

To examine behavioral responses going from the open‐field test to the mirror test, we analyzed activity and acceleration from the 3‐month trials (45 cod and 16 saithe), now including a species‐comparison mixed‐effects model. For each response variable, we fitted a Gaussian Bayesian mixed model with fixed effects of species, context (open‐field vs. mirror), and their interaction (species × context). To quantify among‐individual variation within species, we fitted species‐specific random intercepts and random slopes across context for all individuals using a grouped random‐effects structure (1 + context | gr(ID, by = species)). This allowed both behavior and context‐dependent plasticity to vary among individuals within each species. Residual variance was allowed to differ between species (sigma ~0 + species).

#### 2.6.3 Transition From Solitary to Paired Tests (Model 3)

Finally, a model identical to model 2 was used to estimate species‐specific behavioral responses as the fish transitioned from solitary to paired trials. The paired trials included only a subset of fish (18 cod and 14 saithe). Both solitary and paired trials included open‐field and mirror tests in equal numbers. Context was modeled as a two‐level factor (solitary; paired), and species differences were assessed using a species × context interaction. As explained above, paired trials followed the third (and final) monthly trial, as it was logistically impossible to conduct pairing based on size differences across development and different growth rates. Thus, context effects from this model should be interpreted together with other trait changes across the 3 months including growth and development.

## 3 Results

The standard growth rate (SGR) across the 3 months was 1.37% ± 0.4% day^−1^ for cod, and for saithe, 1.36% ± 0.4% day^−1^. A single cod died after 2 months, but the data for the first two measures were included in the analysis. In the 3‐month solitary trials, 268 observations of 45 Atlantic cod (observations for one fish were partly missing) and 96 observations of 16 saithe were available, while in the paired trials, 18 cod (108 observations) and 14 saithe (84 observations) were available. Species differed significantly but modestly in acceleration and latency to explore, but not in other traits (Figure 2) and movement traits were more strongly coupled in saithe (Figure 3). Full posterior estimates, 95% credible intervals, and variance components for all models are provided in the Appendix S1. Unconditional repeatability (ICC) for activity was low for cod at 0.01 (95% CI: 0.00–0.06) and moderate at 0.24 (95% CI: 0.10–0.45) for acceleration; for saithe, repeatability was moderate at 0.25 (95% CI: 0.09–0.48) for activity and low at 0.03 (95% CI: 0.00–0.12) for acceleration.

### 3.1 State Dependent Variation in Movement Traits Across Time (Model 1)

**FIGURE 4:**
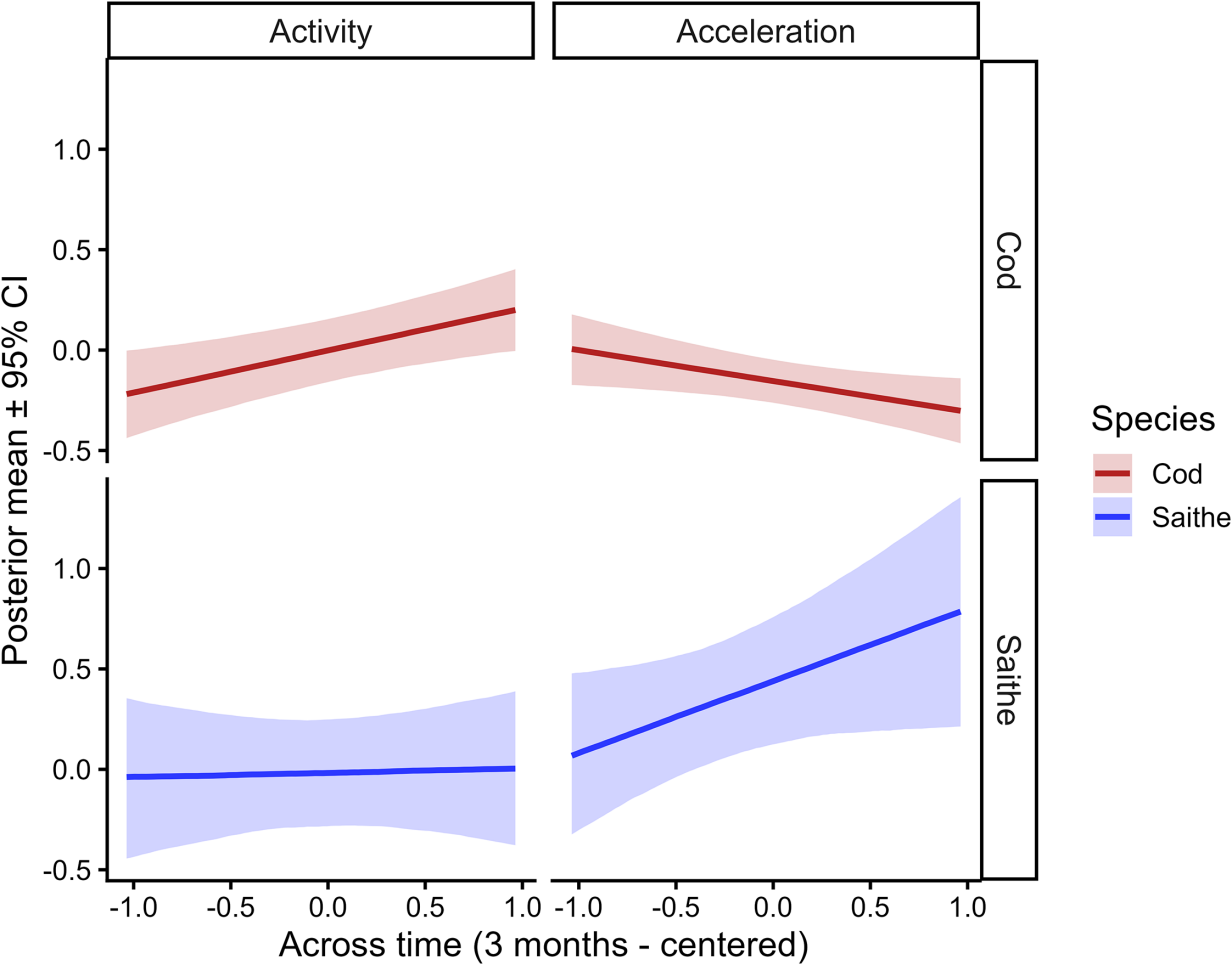
Temporal change in movement traits. Posterior mean and 95% credible intervals showing changes in activity and acceleration across trials for Atlantic cod and saithe. Lines represent species‐level estimates from multivariate Bayesian mixed‐effects models.

For Atlantic cod, acceleration decreased slightly across the 3‐month period (Estimate = −0.15, 95% CI: −0.27 to −0.03) but activity increased slightly (Estimate = 0.21, 95% CI: 0.07–0.35). In saithe, the posterior estimates were uncertain. Trends across time are shown in Figure 4. For cod, acceleration was slightly but positively associated with body length; although the estimate was low, the credible intervals suggest it is robust (Estimate = 0.17, 95% CI: 0.10–0.24). No other state variables were supported for either cod or saithe. Among‐individual variation was moderate for cod activity (Estimate = 0.36, 95% CI: 0.18–0.55) and low for cod acceleration (Estimate = 0.12, 95% CI: 0.01–0.28). Residual correlation between acceleration and activity was negligible for cod. Conversely, estimates of among‐individual variation were higher for saithe acceleration (Estimate = 0.42, 95% CI: 0.08–0.81) and slope (Estimate = 0.50, 95% CI: 0.10–0.90) but low for activity. In contrast to cod, residual acceleration and activity were positively correlated in saithe (Estimate = 0.32, 95% CI: 0.11–0.59). Saithe also exhibited higher residual variation than cod, indicating greater within‐individual variability after accounting for fixed and random effects.

### 3.2 Transition From Open‐Field to Mirror Test (Model 2)

**FIGURE 5:**
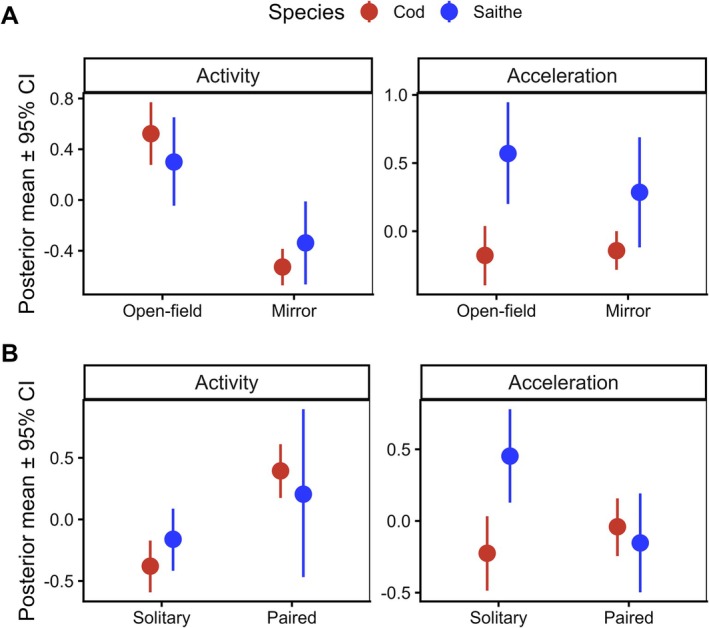
Model‐derived estimates to experimental context. Panel A shows responses from open‐field to mirror trials, and Panel B shows responses from solitary to paired trials. Points and intervals show posterior means and 95% credible intervals for context‐specific predicted values reconstructed from the fitted Bayesian mixed‐effects models.

Exposure to a mirror reduced activity of both species (Estimate = −1.06, 95% CI: −1.29 to −0.82) (Figure 5). Baseline activity did not differ clearly between species, and the species × context interaction was uncertain. Among‐individual variation in baseline activity was very high in cod (Estimate = 0.76, 95% CI: 0.58–0.98) and lower in saithe (Estimate = 0.32, 95% CI: 0.02–0.78), with large uncertainty around the saithe estimate. Similarly, cod showed considerable among‐individual variation in mirror responsiveness (Estimate = 0.60, 95% CI: 0.40–0.84), while among‐individual variation in saithe was both lower and less precisely estimated (Estimate = 0.23, 95% CI: 0.01–0.69). In cod, intercept–slope correlations were strongly negative for activity (Estimate = −0.90, 95% CI: −0.99 to −0.75). No clear intercept–slope correlation was shown for saithe.

In contrast to activity, acceleration showed no clear effect of mirror exposure across species and there were no species differences in response to the mirror (Figure 5). However, saithe had much higher baseline acceleration than cod (Estimate = 0.75, 95% CI: 0.32–1.17). Among‐individual variation in acceleration was high for cod (Estimate = 0.60, 95% CI: 0.41–0.82) but lower for saithe with broad credible intervals (Estimate = 0.25, 95% CI: 0.01–0.67). Cod again showed substantial among‐individual variation in mirror responsiveness (Estimate = 0.50, 95% CI: 0.26–0.75), while saithe estimates were uncertain. Intercept–slope correlations were again strongly negative in cod (Estimate = −0.96, 95% CI: −0.99 to −0.84), indicating that individuals with higher baseline acceleration reduced their acceleration more strongly under mirror exposure. No clear correlation was shown for saithe. Residual variation also differed between species; saithe exhibited higher within‐individual variability than cod.

### 3.3 Transition From Solitary to Paired Tests (Model 3)

Activity increased substantially when individuals were tested in paired contexts compared to solitary trials (Estimate = 0.77, 95% CI: 0.41–1.13) (Figure 5). Baseline activity in solitary trials did not differ clearly between species (Estimate = 0.22, 95% CI: −0.10–0.53). Among‐individual variation in activity response across contexts was high in both species (Estimates: cod = 0.64, 95% CI: 0.36–1.02; saithe = 1.13, 95% CI: 0.67–1.82), while among‐individual variation in activity was moderate for cod (Estimate = 0.34, 95% CI: 0.16–0.57) and low to moderate for saithe with broad credible intervals (Estimate = 0.23, 95% CI: 0.01–0.58). For cod, intercept–slope correlations were again strongly negative (Estimate = −0.91, 95% CI: −1.00 to −0.63), indicating that individuals with lower baseline activity in solitary trials exhibited stronger increases when paired. No clear intercept–slope correlation was detected in saithe.

For cod, the model did not support a clear change in acceleration from solitary to paired trials (Figure 5). Again, saithe exhibited higher acceleration than cod in solitary trials (Estimate = 0.68, 95% CI: 0.23–1.09), and the species × context interaction was strongly negative (Estimate = −0.79, 95% CI: −1.32 to −0.28), indicating that saithe reduced acceleration more in paired trials relative to cod. Among‐individual variation was moderate to high in cod (Estimate = 0.48, 95% CI: 0.29–0.73) but lower with broader credible intervals in saithe (Estimate = 0.32, 95% CI: 0.02–0.79). Among‐individual variation in response was moderate in cod (Estimate = 0.31, 95% CI: 0.02–0.64) and saithe (Estimate = 0.42, 95% CI: 0.02–1.05). Intercept–slope correlations were weak and uncertain in both species. Saithe again exhibited higher residual variation.

## 4 Discussion

The two species differed modestly but consistently in behavioral expression (Figure 2). Saithe appeared more reactive, leaving the release chamber more quickly and showing higher acceleration, whereas Atlantic cod were slower to explore and showed less burst‐like movement. Beyond these mean differences, behavioral individuality was evident in both species, but individual responses were more strongly structured in Atlantic cod. This contrast aligns with known ecological differences between the species. Juvenile cod show divergent movement strategies, from broad migrations (Pálsson 1976) to territoriality (Tupper and Boutilier 1995), and tend to shelter in response to risk (Valliant et al. 2025). Although fewer studies have examined juvenile saithe movement, adult saithe are highly shoaling, migratory, and prolonged swimmers (He and Wardle 1988; Homrum et al. 2012, 2013; Myksvoll et al. 2021). Recent field‐based comparisons indicate that YOY Atlantic cod show facultative persistence in coastal nurseries, whereas same‐aged saithe occur more sporadically in opportunistic shoals (Ólafsdóttir and Nickel 2026). Thus, the greater reactivity, conspecific response, and higher residual variability observed in saithe, together with the slower exploration and more structured individual responses observed in Atlantic cod, are broadly consistent with expectations based on cod–saithe ecology. These results highlight that analyses of behavioral structure of non‐model species can provide insight into links between behavior and ecology or life‐history variation.

Cod increased activity notably across time while acceleration declined and this was consistent with adjustment to the experimental setting but could also indicate developmental changes over the 3‐month period. Larger Atlantic cod had slightly higher acceleration, but there were no other supported effects of length, condition, or growth rate for either species. This limited evidence for state effects is noteworthy as relative body size as well as early growth rate are known to have important consequences for outcomes of YOY fish (Post and Evans 1989; Ólafsdóttir et al. 2015) and may affect life‐history strategies, for example as cod juveniles in different condition adopt different residencies or have different survival likelihoods (Geissinger et al. 2021; Ólafsdóttir and Nickel 2026). However, other studies have also found mixed relationships between laboratory estimates of behavior, individual size, and field‐based metrics in early juveniles (Näslund et al. 2017; Ladurée et al. 2026), suggesting considerable stochasticity or environmental dependence. The current results show that both time and body size did affect cod but not saithe behavior, which is consistent with saithe movement being more labile and cod more state dependent or facultative. Previous studies have shown effects of temperature, growth, and size on juvenile cod swimming (Martínez et al. 2004; Peck et al. 2006). However, it should be noted that the experimental cod had a broader size range which could explain why the relationship is only apparent for cod.

Context had strong but contrasting effects on juvenile activity. In both species, activity was markedly reduced when the mirror was present, with no clear difference in magnitude between species. However, in paired trials, the effect was reversed, and both species increased activity relative to solitary trials. Acceleration response also diverged between species. Cod maintained similar acceleration across solitary and paired contexts, whereas saithe exhibited reduced acceleration when paired. Although the paired trials followed the solitary trials, growth or development alone is unlikely to fully explain this pattern. This is particularly clear for saithe, where there was no detectable change in trait expression over the 3‐month period but a strong shift between solitary and paired trials. Timing alone therefore seems insufficient to account for the observed differences. The contrasting responses to the two contexts likely reflect differences in how juveniles experienced the mirror versus a live conspecific. The mirror test is frequently used as a social cue in fish behavioral ecology, for example, to evaluate sociability (Beukeboom et al. 2023) or aggression (Teles and Oliveira 2016). However, other studies have suggested that a mirror image may not reflect social cues (Budaev 1997) and found discrepancies in responses when exposed to a mirror versus in the presence of a conspecific (Cattelan et al. 2017). Other behavioral studies have used visual exposure to live conspecifics (Ólafsdóttir and Magellan 2016) or included a mirror observable at a distance (Cattelan et al. 2017) with more comparable results to exposure to a live conspecific. While the mirror test has several advantages (Rowland 1999), including logistic feasibility and standardization of the stimuli relative to the tested fish, the current results suggest that it increased vigilance or hesitancy, in contrast to interaction with a live conspecific, and was less appropriate as a social context.

Beyond mean differences, the species differed in how behavioral variation was structured among individuals and within individuals. Among‐individual variation across time was evident in both species, but the organization of that variation differed. Coupled to the more directional population level change in activity, cod showed only moderate individuality in activity over the 3 months whereas saithe showed consistent among‐individual differences only in acceleration. Species estimates of unconditional repeatability were parallel to the estimates of among‐individual variation across the 3 months. This suggests that when context is not considered and behavior is measured over longer periods of time, behavioral consistency of these juvenile fish was not consistent across all movement traits. However, activity and acceleration were positively correlated at the residual level for saithe, suggesting a tighter coupling of within‐individual movement traits across development. Cod, in contrast, exhibited no coupling between these traits. Young fish, larvae, and YOY juveniles inevitably shift their locomotion or swimming dynamics across development, and this has been shown to be affected by size (Webb and Weihs 1986; Leis 2010). The current study represents a relatively short period for morphologically similar juveniles that have reached the size for stable settlement to nursery grounds (Ólafsdóttir and Nickel 2026), and this may reflect the slight size‐ or time‐related variation observed in the current data.

**FIGURE 6:**
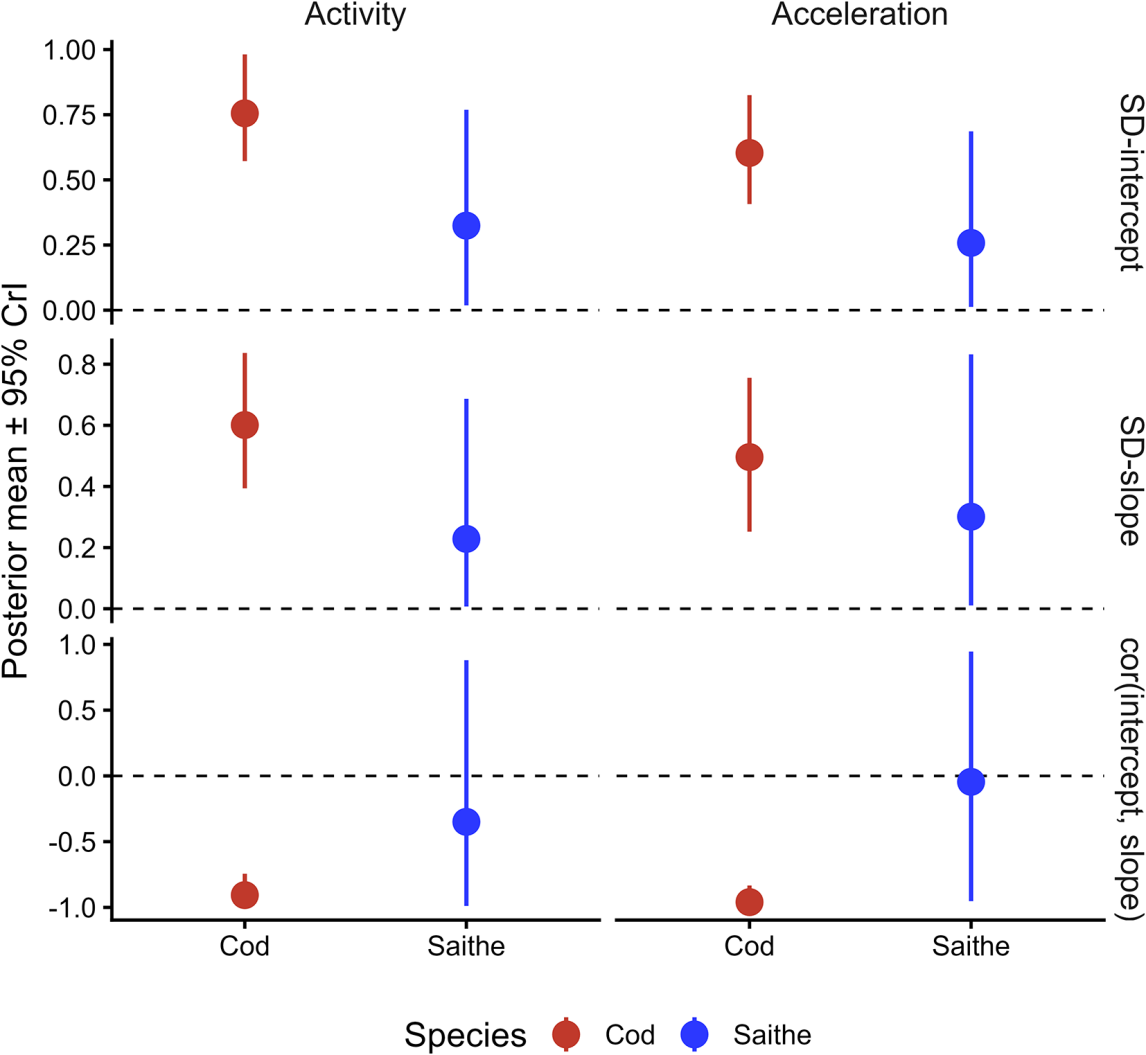
Species‐specific individual‐level variation components for movement responses across open‐field to mirror context. Posterior means and 95% credible intervals are shown for among‐individual baseline variation (intercept), among‐individual variation in context‐dependent plasticity (slope), and intercept–slope correlations.

Cod exhibited higher individuality than saithe across contexts and very strong intercept–slope relationships that were consistent across both mirror and paired contexts (Figure 6). The intercept‐slope relationship is important as it shows that Atlantic cod individuals differed predictably in how their baseline expression of a trait resulted in response in the same trait, that is, their behavioral plasticity. Saithe showed only moderate individuality, higher residual variation, and comparatively less stable structured responses across contexts. The current results add to the accumulating evidence of individuality in behavioral expression in early life‐stages (Näslund et al. 2017; de Bivort 2025) as behavioral plasticity was found to be an important component of individuality, particularly in the Atlantic cod. This is consistent with behavioral reaction norm frameworks, which show that context‐dependent responses are an integral component of animal personality expression (e.g., Dingemanse et al. 2010; Dingemanse and Dochtermann 2013). However, our results also indicated that behavioral structure differs among even related, ecologically similar species, though these results should be interpreted conservatively due to the lower sample size for saithe. Specifically, across the models in the current study the saithe estimates were relatively more uncertain, and while this can reflect the inherently more labile nature of saithe movement it can also be an artifact of the lower sample size for saithe. However, the same contrast was also evident in the paired‐trial models, where sample sizes were more balanced between species, suggesting that the stronger structure of cod reaction norms was not solely an artifact of lower saithe sample size.

Trait correlations further highlight these species differences. Movement traits tended to be more strongly coupled in saithe than in cod at both among‐ and within‐individual levels. In saithe, individuals that were active for longer durations also tended to show greater acceleration, and this tighter clustering extended to other movement traits measured in the study. In cod, correlations were weak, inconsistent, or absent, indicating that even traits integral to movement and swimming can be loosely structured in some species. This loose coupling may facilitate facultative or state‐dependent behavioral responses in heterogeneous environments, whereas the tighter integration observed in saithe may reflect evolutionary or biomechanical constraints associated with shoaling and coordinated swimming. Previous studies have highlighted that behavioral flexibility can contribute to species evolvability and resilience (Dellinger et al. 2025). The current results highlight that context has the potential to result in very different behavioral responses of related, co‐existing species. Although the present results do not establish causal links between the laboratory behavior and field outcomes, they show high levels of structured individuality, structured plasticity, and loose trait coupling in Atlantic cod, compared with more integrated, but labile, and socially responsive movement in saithe. These behavioral components could plausibly affect field outcomes such as foraging, predator avoidance, habitat use, and even survival, although direct tests will require controlled field studies, for example using individual tagging that could allow precise estimates of behavior across field conditions.

To summarize, this study shows that two closely related and ecologically overlapping gadoids differ not only in average movement expression, but also in the organization of behavioral variation during early life. Mean species differences were modest: saithe were quicker to explore and showed higher speed‐corrected acceleration, whereas cod showed clearer temporal and size‐related adjustment in movement traits. The stronger contrast was in behavioral structure. Cod showed more consistently structured individual reaction norms, including baseline links to plasticity and relatively weak coupling among movement traits, suggesting that behavioral traits can vary more independently for Atlantic cod. In contrast, saithe showed tighter integration among movement traits, higher residual variability, and reduced acceleration relative to cod in paired trials, consistent with a more integrated and socially responsive movement mode. These laboratory assays do not reproduce the complexity of natural nursery habitats, but they isolate components of movement behavior that are difficult to separate clearly in the field: state dependence, context‐dependent plasticity, individual reaction norms, and trait coupling. The resulting patterns align with ecological contrasts between facultatively resident, behaviorally heterogeneous Atlantic cod and more shoaling, mobile saithe. Thus, the current results suggest that early life behavioral structure may contribute to divergent movement strategies later in life.

## Author Contributions

**Guðbjörg Ásta Ólafsdóttir:** conceptualization (equal), data curation (lead), formal analysis (lead), funding acquisition (lead), writing – original draft (lead), writing – review and editing (equal). **Michelle L. Valliant:** conceptualization (equal), investigation (lead), methodology (lead), project administration (lead), writing – review and editing (equal).

## Funding

This work was supported by The Icelandic Research Fund, grant number 195876.

## Conflicts of Interest

The authors declare no conflicts of interest.

## Supporting information


**Appendix S1:** Full model output.

**Table S1:** Data used in the paper.

## Data Availability

The data used in this paper can be found in Table S1.
