# Supplementary material for: Young‐Of‐Year Atlantic Cod and Saithe Differ in Individuality and Structure of Movement Traits
Source: Ecol Evol. 2026 Aug 2;16(8):e74149. doi: 10.1002/ece3.74149 (PMC13429882; doi:10.1002/ece3.74149)
Supplement: Supplementary file 1 — Appendix S1: Full model output. [file ECE3-16-e74149-s002.docx]

**APPENDIX**

Table A1. Full model output.

| **Model** | **Response** | **Species** | **Component** | **Term** | **Estimate** | **CI_low** | **CI_high** | **Rhat** | **Bulk_ESS** | **Tail_ESS** |
| --- | --- | --- | --- | --- | --- | --- | --- | --- | --- | --- |
| Model 1 | Acceleration | Cod | Fixed effect | Growth rate | -0.43 | -1.02 | 0.16 | 1.00 | 17171 | 8923 |
| Model 1 | Activity | Cod | Fixed effect | Growth rate | 0.62 | -0.24 | 1.46 | 1.00 | 9835 | 9094 |
| Model 1 | Acceleration | Saithe | Fixed effect | Growth rate | 0.58 | -0.58 | 1.73 | 1.00 | 10788 | 8990 |
| Model 1 | Activity | Saithe | Fixed effect | Growth rate | -0.22 | -1.28 | 0.84 | 1.00 | 12906 | 9367 |
| Model 1 | Activity | Cod | Random structure | ID correlation: intercept-slope | -0.16 | -0.97 | 0.91 | 1.00 | 11745 | 7137 |
| Model 1 | Acceleration | Saithe | Random structure | ID correlation: intercept-slope | 0.72 | -0.23 | 1.00 | 1.00 | 4805 | 4199 |
| Model 1 | Activity | Saithe | Random structure | ID correlation: intercept-slope | -0.07 | -0.96 | 0.94 | 1.00 | 14613 | 8693 |
| Model 1 | Acceleration | Cod | Random structure | ID correlation: intercept-slope | -0.03 | -0.96 | 0.94 | 1.00 | 12920 | 8297 |
| Model 1 | Acceleration | Cod | Random structure | ID SD: intercept | 0.13 | 0.01 | 0.30 | 1.00 | 3325 | 4961 |
| Model 1 | Activity | Cod | Random structure | ID SD: intercept | 0.37 | 0.18 | 0.56 | 1.00 | 2979 | 2595 |
| Model 1 | Acceleration | Saithe | Random structure | ID SD: intercept | 0.42 | 0.08 | 0.81 | 1.00 | 3840 | 3286 |
| Model 1 | Activity | Saithe | Random structure | ID SD: intercept | 0.25 | 0.01 | 0.64 | 1.00 | 3781 | 6300 |
| Model 1 | Acceleration | Cod | Random structure | ID SD: slope | 0.09 | 0.00 | 0.24 | 1.00 | 6579 | 6617 |
| Model 1 | Activity | Cod | Random structure | ID SD: slope | 0.10 | 0.00 | 0.28 | 1.00 | 5929 | 5773 |
| Model 1 | Acceleration | Saithe | Random structure | ID SD: slope | 0.50 | 0.10 | 0.94 | 1.00 | 4106 | 3108 |
| Model 1 | Activity | Saithe | Random structure | ID SD: slope | 0.16 | 0.01 | 0.48 | 1.00 | 8078 | 6374 |
| Model 1 | Acceleration | Cod | Fixed effect | Intercept | -0.79 | -1.65 | 0.08 | 1.00 | 21064 | 9965 |
| Model 1 | Activity | Cod | Fixed effect | Intercept | 0.62 | -0.63 | 1.88 | 1.00 | 11323 | 9459 |
| Model 1 | Acceleration | Saithe | Fixed effect | Intercept | -1.08 | -7.02 | 4.80 | 1.00 | 12635 | 9240 |
| Model 1 | Activity | Saithe | Fixed effect | Intercept | 0.92 | -4.61 | 6.63 | 1.00 | 14593 | 8796 |
| Model 1 | Acceleration | Cod | Fixed effect | K | -16.66 | -94.39 | 60.68 | 1.00 | 17823 | 9978 |
| Model 1 | Activity | Cod | Fixed effect | K | -53.63 | -166.31 | 58.97 | 1.00 | 10992 | 9421 |
| Model 1 | Acceleration | Saithe | Fixed effect | K | -3.75 | -406.29 | 395.64 | 1.00 | 13390 | 9822 |
| Model 1 | Activity | Saithe | Fixed effect | K | 7.38 | -362.29 | 375.75 | 1.00 | 15567 | 8998 |
| Model 1 | Acceleration | Cod | Fixed effect | Length | 0.17 | 0.11 | 0.24 | 1.00 | 16117 | 8748 |
| Model 1 | Activity | Cod | Fixed effect | Length | -0.06 | -0.17 | 0.04 | 1.00 | 9345 | 9116 |
| Model 1 | Acceleration | Saithe | Fixed effect | Length | 0.17 | -0.35 | 0.67 | 1.00 | 10613 | 9126 |
| Model 1 | Activity | Saithe | Fixed effect | Length | -0.12 | -0.60 | 0.35 | 1.00 | 13689 | 9830 |
| Model 1 | Activity-acceleration | Cod | Random structure | Residual correlation | -0.01 | -0.14 | 0.12 | 1.00 | 19790 | 8617 |
| Model 1 | Activity-acceleration | Saithe | Random structure | Residual correlation | 0.32 | 0.11 | 0.50 | 1.00 | 13940 | 8662 |
| Model 1 | Acceleration | Cod | Random structure | Residual SD | 0.81 | 0.74 | 0.88 | 1.00 | 16553 | 8655 |
| Model 1 | Acceleration | Saithe | Random structure | Residual SD | 1.09 | 0.92 | 1.28 | 1.00 | 8622 | 7881 |
| Model 1 | Activity | Cod | Random structure | Residual SD | 0.89 | 0.81 | 0.98 | 1.00 | 9517 | 8813 |
| Model 1 | Activity | Saithe | Random structure | Residual SD | 1.09 | 0.94 | 1.27 | 1.00 | 15591 | 9710 |
| Model 1 | Acceleration | Cod | Fixed effect | TRIAL_c | -0.15 | -0.28 | -0.02 | 1.00 | 23876 | 8541 |
| Model 1 | Activity | Cod | Fixed effect | TRIAL_c | 0.21 | 0.07 | 0.35 | 1.00 | 26458 | 8338 |
| Model 1 | Acceleration | Saithe | Fixed effect | TRIAL_c | 0.36 | -0.04 | 0.74 | 1.00 | 10201 | 8779 |
| Model 1 | Activity | Saithe | Fixed effect | TRIAL_c | 0.02 | -0.26 | 0.31 | 1.00 | 18684 | 8114 |
| Model 2 | Activity | Cod | Random structure | ID correlation: intercept-slope | -0.91 | -0.99 | -0.74 | 1.00 | 3929 | 3577 |
| Model 2 | Activity | Saithe | Random structure | ID correlation: intercept-slope | -0.35 | -0.99 | 0.88 | 1.00 | 7654 | 7235 |
| Model 2 | Acceleration | Cod | Random structure | ID correlation: intercept-slope | -0.96 | -1.00 | -0.83 | 1.00 | 9044 | 8022 |
| Model 2 | Acceleration | Saithe | Random structure | ID correlation: intercept-slope | -0.05 | -0.95 | 0.95 | 1.00 | 11829 | 9050 |
| Model 2 | Activity | Cod | Random structure | ID SD: intercept | 0.76 | 0.57 | 0.98 | 1.00 | 3329 | 5889 |
| Model 2 | Activity | Saithe | Random structure | ID SD: intercept | 0.32 | 0.02 | 0.77 | 1.00 | 3109 | 4643 |
| Model 2 | Acceleration | Cod | Random structure | ID SD: intercept | 0.60 | 0.41 | 0.83 | 1.00 | 3787 | 4711 |
| Model 2 | Acceleration | Saithe | Random structure | ID SD: intercept | 0.26 | 0.01 | 0.69 | 1.00 | 5249 | 5934 |
| Model 2 | Activity | Cod | Random structure | ID SD: slope | 0.60 | 0.39 | 0.84 | 1.00 | 4690 | 6315 |
| Model 2 | Activity | Saithe | Random structure | ID SD: slope | 0.23 | 0.01 | 0.69 | 1.00 | 5285 | 5525 |
| Model 2 | Acceleration | Cod | Random structure | ID SD: slope | 0.50 | 0.25 | 0.76 | 1.00 | 5379 | 4474 |
| Model 2 | Acceleration | Saithe | Random structure | ID SD: slope | 0.30 | 0.01 | 0.83 | 1.00 | 5722 | 6177 |
| Model 2 | Activity | Both species | Fixed effect | Intercept | 0.52 | 0.28 | 0.77 | 1.00 | 2567 | 5317 |
| Model 2 | Acceleration | Both species | Fixed effect | Intercept | -0.18 | -0.40 | 0.04 | 1.00 | 5267 | 8477 |
| Model 2 | Activity | Cod | Random structure | Residual SD, log scale | -0.50 | -0.60 | -0.40 | 1.00 | 7586 | 7996 |
| Model 2 | Acceleration | Cod | Random structure | Residual SD, log scale | -0.27 | -0.37 | -0.18 | 1.00 | 11453 | 8715 |
| Model 2 | Activity | Saithe | Random structure | Residual SD, log scale | 0.00 | -0.15 | 0.16 | 1.00 | 11925 | 9058 |
| Model 2 | Acceleration | Saithe | Random structure | Residual SD, log scale | 0.16 | 0.01 | 0.32 | 1.00 | 19928 | 9161 |
| Model 2 | Activity | Both species | Fixed effect | SpeciesSaithe | -0.22 | -0.65 | 0.21 | 1.00 | 5263 | 7694 |
| Model 2 | Acceleration | Both species | Fixed effect | SpeciesSaithe | 0.75 | 0.32 | 1.18 | 1.00 | 8491 | 9579 |
| Model 2 | Activity | Both species | Fixed effect | SpeciesSaithe:TESTmirror | 0.41 | -0.08 | 0.90 | 1.00 | 7463 | 9055 |
| Model 2 | Acceleration | Both species | Fixed effect | SpeciesSaithe:TESTmirror | -0.32 | -0.87 | 0.22 | 1.00 | 10728 | 9810 |
| Model 2 | Activity | Both species | Fixed effect | TESTmirror | -1.05 | -1.29 | -0.82 | 1.00 | 4059 | 6547 |
| Model 2 | Acceleration | Both species | Fixed effect | TESTmirror | 0.03 | -0.20 | 0.27 | 1.00 | 7394 | 9081 |
| Model 3 | Activity | Both species | Fixed effect | ContextPaired | 0.77 | 0.41 | 1.13 | 1.00 | 6784 | 7508 |
| Model 3 | Acceleration | Both species | Fixed effect | ContextPaired | 0.18 | -0.04 | 0.41 | 1.00 | 5417 | 7331 |
| Model 3 | Activity | Cod | Random structure | ID correlation: intercept-slope | -0.91 | -1.00 | -0.64 | 1.00 | 4900 | 7734 |
| Model 3 | Activity | Saithe | Random structure | ID correlation: intercept-slope | 0.10 | -0.81 | 0.94 | 1.00 | 1664 | 2813 |
| Model 3 | Acceleration | Cod | Random structure | ID correlation: intercept-slope | -0.73 | -0.99 | -0.01 | 1.00 | 6101 | 6169 |
| Model 3 | Acceleration | Saithe | Random structure | ID correlation: intercept-slope | -0.50 | -0.99 | 0.81 | 1.00 | 4590 | 6818 |
| Model 3 | Activity | Cod | Random structure | ID SD: intercept | 0.34 | 0.16 | 0.57 | 1.00 | 5079 | 6108 |
| Model 3 | Activity | Saithe | Random structure | ID SD: intercept | 0.23 | 0.01 | 0.58 | 1.00 | 4306 | 5211 |
| Model 3 | Acceleration | Cod | Random structure | ID SD: intercept | 0.48 | 0.29 | 0.73 | 1.00 | 4249 | 6811 |
| Model 3 | Acceleration | Saithe | Random structure | ID SD: intercept | 0.32 | 0.02 | 0.79 | 1.00 | 2624 | 4396 |
| Model 3 | Activity | Cod | Random structure | ID SD: slope | 0.64 | 0.36 | 1.01 | 1.00 | 4411 | 6914 |
| Model 3 | Activity | Saithe | Random structure | ID SD: slope | 1.13 | 0.67 | 1.84 | 1.00 | 4741 | 7729 |
| Model 3 | Acceleration | Cod | Random structure | ID SD: slope | 0.31 | 0.05 | 0.60 | 1.00 | 3784 | 3199 |
| Model 3 | Acceleration | Saithe | Random structure | ID SD: slope | 0.42 | 0.02 | 1.05 | 1.00 | 2930 | 5276 |
| Model 3 | Activity | Both species | Fixed effect | Intercept | -0.38 | -0.59 | -0.17 | 1.00 | 7276 | 7754 |
| Model 3 | Acceleration | Both species | Fixed effect | Intercept | -0.23 | -0.49 | 0.03 | 1.00 | 3283 | 5490 |
| Model 3 | Activity | Cod | Random structure | Residual SD, log scale | -0.40 | -0.50 | -0.29 | 1.00 | 14213 | 8782 |
| Model 3 | Acceleration | Cod | Random structure | Residual SD, log scale | -0.47 | -0.58 | -0.37 | 1.00 | 12208 | 8821 |
| Model 3 | Activity | Saithe | Random structure | Residual SD, log scale | -0.09 | -0.21 | 0.03 | 1.00 | 14516 | 8848 |
| Model 3 | Acceleration | Saithe | Random structure | Residual SD, log scale | 0.18 | 0.07 | 0.30 | 1.00 | 10107 | 8560 |
| Model 3 | Activity | Both species | Fixed effect | SpeciesSaithe | 0.22 | -0.11 | 0.54 | 1.00 | 8653 | 8517 |
| Model 3 | Acceleration | Both species | Fixed effect | SpeciesSaithe | 0.68 | 0.27 | 1.09 | 1.00 | 4896 | 6770 |
| Model 3 | Activity | Both species | Fixed effect | SpeciesSaithe:ContextPaired | -0.41 | -1.19 | 0.36 | 1.00 | 6525 | 7695 |
| Model 3 | Acceleration | Both species | Fixed effect | SpeciesSaithe:ContextPaired | -0.79 | -1.31 | -0.28 | 1.00 | 6640 | 7981 |
