# Supplementary material for: Young‐Of‐Year Atlantic Cod and Saithe Differ in Individuality and Structure of Movement Traits
Source: Ecol Evol. 2026 Aug 2;16(8):e74149. doi: 10.1002/ece3.74149 (PMC13429882; doi:10.1002/ece3.74149)
Supplement: Supplementary file 2 — Table S1: Data used in the paper. [file ECE3-16-e74149-s001.docx]

**Young-of-year Atlantic cod and saithe differ in individuality and structure of movement traits**

Guðbjörg Ásta Ólafsdóttir^1,*^ & Michelle L. Valliant^1^

^1^University of Iceland, Research Centre of the Westfjords, Adalstræti 10-12, 415 Bolungarvik, Iceland

*Corresponding Author: Guðbjörg Ásta Ólafsdóttir

Email address: gaol@hi.is

**Supplementary table 1.** Data used in the paper.

| YEAR | TRIAL | ID | LE | Visits | Active | Distance | Speed | Acceleration | TEST | Length | CONTEXT |
| --- | --- | --- | --- | --- | --- | --- | --- | --- | --- | --- | --- |
| 2020 | 3 | C6 | 58 | 5 | 507.26 | 1958.3 | 3.52 | 86.47 | open-field | 10.01 | Solitary |
| 2020 | 3 | C8 | 46 | 22 | 535.51 | 3643.18 | 6.98 | 144.76 | open-field | 10.41 | Solitary |
| 2020 | 3 | C10 | 275 | 36 | 577.82 | 4605.13 | 8.81 | 147.21 | open-field | 4.91 | Solitary |
| 2020 | 3 | C11 | 60 | 43 | 563.41 | 3621.72 | 6.62 | 111.81 | open-field | 6.39 | Solitary |
| 2020 | 3 | C12 | 180 | 19 | 304.11 | 1452.7 | 3.7 | 74.57 | open-field | 5.97 | Solitary |
| 2020 | 3 | C13 | 60 | 5 | 315.98 | 964.4 | 0.85 | 7.8 | open-field | 8.23 | Solitary |
| 2020 | 3 | C14 | 230 | 17 | 517.05 | 2822.25 | 4.08 | 35.69 | open-field | 5.99 | Solitary |
| 2020 | 3 | C15 | 0 | 17 | 471.18 | 2132.52 | 5.38 | 114.3 | open-field | 6.43 | Solitary |
| 2020 | 3 | C16 | 300 | 2 | 473.83 | 3343.27 | 3.9 | 77.1 | open-field | 8.17 | Solitary |
| 2020 | 3 | C17 | 300 | 42 | 412.32 | 2933.2 | 4.54 | 53.55 | open-field | 5.14 | Solitary |
| 2020 | 3 | S1 | 24 | 71 | 540.03 | 5417.57 | 6.65 | 66.05 | open-field | 6.28 | Solitary |
| 2020 | 3 | S2 | 0 | 69 | 585.09 | 6138.01 | 5.62 | 54.24 | open-field | 6.27 | Solitary |
| 2020 | 3 | S3 | 0 | 14 | 154.56 | 1285.46 | 3.97 | 38.44 | open-field | 7.91 | Solitary |
| 2020 | 3 | S4 | 300 | 32 | 456.44 | 1963.99 | 5.36 | 75.38 | open-field | 6.72 | Solitary |
| 2020 | 3 | S5 | 300 | 32 | 571.11 | 4412.58 | 5.12 | 51.92 | open-field | 7.59 | Solitary |
| 2020 | 3 | C2 | 0 | 18 | 489.55 | 2316.34 | 8.16 | 152.27 | open-field | 6.4 | Solitary |
| 2020 | 3 | C3 | 0 | 1 | 466.39 | 1442.14 | 1.29 | 28.65 | open-field | 5.56 | Solitary |
| 2020 | 3 | C4 | 300 | 2 | 508.8 | 1916.8 | 1.67 | 36.42 | open-field | 6.37 | Solitary |
| 2020 | 3 | C5 | 0 | 5 | 568.59 | 4587.78 | 15.44 | 346.83 | open-field | 10.45 | Solitary |
| 2020 | 3 | S8 | 300 | 1 | 563.34 | 20467.26 | 13.26 | 429.65 | open-field | 7.94 | Solitary |
| 2020 | 3 | S9 | 0 | 5 | 497.82 | 10423.25 | 6.98 | 199.1 | open-field | 7.01 | Solitary |
| 2020 | 3 | S10 | 300 | 19 | 554.82 | 3539.06 | 5.31 | 118.84 | open-field | 6.86 | Solitary |
| 2020 | 3 | S11 | 300 | 31 | 580.17 | 5221.61 | 4.7 | 79.68 | open-field | 7.14 | Solitary |
| 2020 | 3 | S12 | 300 | 67 | 532.01 | 5344.29 | 6.53 | 133.46 | open-field | 7.73 | Solitary |
| 2020 | 3 | S13 | 300 | 25 | 573.85 | 3877.4 | 4.83 | 88.91 | open-field | 6.91 | Solitary |
| 2020 | 3 | S14 | 0 | 51 | 570.97 | 3404.55 | 5.47 | 111.25 | open-field | 7.74 | Solitary |
| 2020 | 3 | S16 | 0 | 32 | 377.19 | 3383.52 | 4.91 | 86.08 | open-field | 7.25 | Solitary |
| 2020 | 3 | S17 | 55 | 51 | 463.79 | 5102.06 | 4.43 | 57.74 | open-field | 8.95 | Solitary |
| 2020 | 3 | S6 | 0 | 39 | 580.83 | 4887.42 | 7.56 | 136.28 | open-field | 7.34 | Solitary |
| 2020 | 3 | S7 | 300 | 12 | 548.38 | 2457.83 | 3.85 | 72.89 | open-field | 6.82 | Solitary |
| 2020 | 3 | C9 | 300 | 36 | 577.82 | 4605.13 | 8.81 | 147.21 | open-field | 10.65 | Solitary |
| 2020 | 2 | C1 | 0 | 3 | 381.6 | 852.99 | 0.83 | 11.53 | open-field | 5.28 | Solitary |
| 2020 | 2 | C2 | 0 | 8 | 452.31 | 2275.16 | 8.77 | 144.26 | open-field | 6.4 | Solitary |
| 2020 | 2 | C3 | 300 | 1 | 332.71 | 1379.61 | 0.57 | 15.14 | open-field | 5.56 | Solitary |
| 2020 | 2 | C4 | 0 | 9 | 500.47 | 2790.84 | 7.92 | 135.44 | open-field | 6.37 | Solitary |
| 2020 | 2 | C5 | 300 | 9 | 554.29 | 6305.07 | 5.47 | 152.02 | open-field | 10.45 | Solitary |
| 2020 | 2 | C6 | 0 | 5 | 498.67 | 5326.96 | 17.17 | 540.93 | open-field | 10.01 | Solitary |
| 2020 | 2 | C8 | 40 | 12 | 562.99 | 8421.22 | 10.3 | 309.52 | open-field | 10.41 | Solitary |
| 2020 | 2 | C9 | 300 | 4 | 567.59 | 4009.46 | 6.11 | 81.71 | open-field | 10.65 | Solitary |
| 2020 | 2 | C10 | 0 | 34 | 389.67 | 2036.9 | 2.45 | 45.8 | open-field | 4.91 | Solitary |
| 2020 | 2 | C11 | 0 | 0 | 449.15 | 1245.54 | 0.52 | 11.92 | open-field | 6.39 | Solitary |
| 2020 | 2 | C12 | 291 | 7 | 402.34 | 948.51 | 1.58 | 31.64 | open-field | 5.97 | Solitary |
| 2020 | 2 | C13 | 0 | 4 | 412.62 | 1022.85 | 2.8 | 36.18 | open-field | 8.23 | Solitary |
| 2020 | 2 | C14 | 0 | 14 | 536.5 | 2236.67 | 2.6 | 49.04 | open-field | 5.99 | Solitary |
| 2020 | 2 | C15 | 0 | 29 | 514.48 | 1769.57 | 4.14 | 47 | open-field | 6.43 | Solitary |
| 2020 | 2 | C16 | 0 | 32 | 559.03 | 3570.24 | 6.16 | 74.81 | open-field | 8.17 | Solitary |
| 2020 | 2 | C17 | 0 | 31 | 480.73 | 3310.68 | 5.21 | 89.99 | open-field | 5.14 | Solitary |
| 2020 | 2 | S1 | 0 | 40 | 609.55 | 4873.54 | 5.55 | 103.95 | open-field | 6.28 | Solitary |
| 2020 | 2 | S2 | 10 | 73 | 834.32 | 8253.24 | 7.25 | 122.66 | open-field | 6.27 | Solitary |
| 2020 | 2 | S3 | 0 | 62 | 861.56 | 7624.55 | 6.36 | 129.73 | open-field | 7.91 | Solitary |
| 2020 | 2 | S4 | 0 | 77 | 863.45 | 5773.55 | 3.13 | 75.64 | open-field | 6.72 | Solitary |
| 2020 | 2 | S5 | 0 | 111 | 727.9 | 8318.46 | 8.44 | 172.41 | open-field | 7.59 | Solitary |
| 2020 | 2 | S6 | 0 | 104 | 881.56 | 8512.07 | 7.49 | 128.53 | open-field | 7.34 | Solitary |
| 2020 | 2 | S7 | 0 | 24 | 608.19 | 5128.42 | 11.32 | 230 | open-field | 6.82 | Solitary |
| 2020 | 2 | S8 | 5 | 34 | 753.21 | 5089.51 | 5.96 | 127.7 | open-field | 7.94 | Solitary |
| 2020 | 2 | S9 | 0 | 21 | 711.29 | 4032.12 | 3.71 | 99.95 | open-field | 7.01 | Solitary |
| 2020 | 2 | S10 | 0 | 40 | 657.59 | 3477.64 | 3.58 | 63.95 | open-field | 6.86 | Solitary |
| 2020 | 2 | S11 | 0 | 209 | 743.11 | 8558.75 | 9.17 | 237.63 | open-field | 7.14 | Solitary |
| 2020 | 2 | S12 | 0 | 28 | 567.5 | 2666.61 | 3.46 | 76.3 | open-field | 7.73 | Solitary |
| 2020 | 2 | S13 | 150 | 54 | 581.29 | 5355.06 | 7.58 | 150.73 | open-field | 6.91 | Solitary |
| 2020 | 2 | S14 | 37 | 127 | 938.3 | 7460.26 | 3.81 | 87.71 | open-field | 7.74 | Solitary |
| 2020 | 2 | S16 | 0 | 18 | 479.94 | 3073.88 | 4.66 | 96.27 | open-field | 7.25 | Solitary |
| 2020 | 2 | S17 | 0 | 54 | 631.63 | 4652.46 | 8.29 | 129.79 | open-field | 8.95 | Solitary |
| 2020 | 1 | C1 | 300 | 28 | 461.61 | 1678.06 | 3.07 | 47.23 | open-field | 5.28 | Solitary |
| 2020 | 1 | C2 | 300 | 16 | 533.11 | 1118.51 | 1.14 | 28.87 | open-field | 6.4 | Solitary |
| 2020 | 1 | C3 | 300 | 7 | 446.67 | 981.44 | 0.86 | 23.1 | open-field | 5.56 | Solitary |
| 2020 | 1 | C4 | 300 | 12 | 463.94 | 1507.45 | 1.4 | 29.95 | open-field | 6.37 | Solitary |
| 2020 | 1 | C5 | 300 | 92 | 745 | 3154.33 | 1.73 | 33 | open-field | 10.45 | Solitary |
| 2020 | 1 | C6 | 300 | 12 | 546.72 | 1260.91 | 1.8 | 42.09 | open-field | 10.01 | Solitary |
| 2020 | 1 | C8 | 62 | 52 | 628.72 | 3800.38 | 7.32 | 90.38 | open-field | 10.41 | Solitary |
| 2020 | 1 | C9 | 300 | 144 | 645.22 | 2339.44 | 3.02 | 57.36 | open-field | 10.65 | Solitary |
| 2020 | 1 | C10 | 0 | 20 | 585.89 | 3767.12 | 2.9 | 73.36 | open-field | 4.91 | Solitary |
| 2020 | 1 | C11 | 300 | 13 | 561.17 | 4542.49 | 11.31 | 209.27 | open-field | 6.39 | Solitary |
| 2020 | 1 | C12 | 300 | 6 | 479.44 | 2023.93 | 3.83 | 82.94 | open-field | 5.97 | Solitary |
| 2020 | 1 | C13 | 300 | 10 | 556.39 | 2792.61 | 3.2 | 85.17 | open-field | 8.23 | Solitary |
| 2020 | 1 | C14 | 300 | 2 | 380.17 | 1085.24 | 1.52 | 32.57 | open-field | 5.99 | Solitary |
| 2020 | 1 | C15 | 300 | 27 | 526.39 | 1756.82 | 3.73 | 72.26 | open-field | 6.43 | Solitary |
| 2020 | 1 | C16 | 300 | 83 | 552 | 5690.42 | 8.79 | 99.78 | open-field | 8.17 | Solitary |
| 2020 | 1 | C17 | 0 | 8 | 391.67 | 946.33 | 1.28 | 25.83 | open-field | 5.14 | Solitary |
| 2020 | 1 | S1 | 3 | 92 | 639 | 3781.12 | 5.11 | 95.68 | open-field | 6.28 | Solitary |
| 2020 | 1 | S2 | 215 | 10 | 390.78 | 3473.7 | 4.41 | 75.64 | open-field | 6.27 | Solitary |
| 2020 | 1 | S3 | 300 | 0 | 0 | 0 | 0 | 0 | open-field | 7.91 | Solitary |
| 2020 | 1 | S4 | 0 | 19 | 479.39 | 1883.75 | 2.76 | 53.61 | open-field | 6.72 | Solitary |
| 2020 | 1 | S5 | 0 | 102 | 822 | 5520.49 | 6.38 | 91.63 | open-field | 7.59 | Solitary |
| 2020 | 1 | S6 | 13 | 38 | 569.11 | 2845.83 | 5.46 | 109.15 | open-field | 7.34 | Solitary |
| 2020 | 1 | S7 | 300 | 260 | 790.44 | 9809.31 | 6.07 | 185.92 | open-field | 6.82 | Solitary |
| 2020 | 1 | S8 | 300 | 1 | 551.67 | 1855.02 | 0.72 | 17.94 | open-field | 7.94 | Solitary |
| 2020 | 1 | S9 | 300 | 18 | 544.83 | 1683.92 | 2.04 | 41.93 | open-field | 7.01 | Solitary |
| 2020 | 1 | S10 | 300 | 11 | 565.06 | 2376.9 | 3.81 | 71.12 | open-field | 6.86 | Solitary |
| 2020 | 1 | S11 | 300 | 3 | 541.67 | 2826.1 | 3.75 | 82.94 | open-field | 7.14 | Solitary |
| 2020 | 1 | S12 | 300 | 35 | 624.89 | 2447.27 | 2.74 | 47.42 | open-field | 7.73 | Solitary |
| 2020 | 1 | S13 | 0 | 17 | 539.5 | 2428.14 | 5.56 | 85.54 | open-field | 6.91 | Solitary |
| 2020 | 1 | S14 | 300 | 18 | 568.83 | 2004.59 | 2.8 | 56.82 | open-field | 7.74 | Solitary |
| 2020 | 1 | S16 | 0 | 1 | 479.5 | 1213.84 | 0.48 | 13.26 | open-field | 7.25 | Solitary |
| 2020 | 1 | S17 | 0 | 10 | 461.5 | 1325.61 | 2.96 | 66.01 | open-field | 8.95 | Solitary |
| 2020 | 1 | C1 | 0 | 19 | 125.22 | 1167.38 | 1.05 | 18.26 | mirror | 5.28 | Solitary |
| 2020 | 2 | C1 | 0 | 20 | 385.04 | 684.69 | 0.57 | 15.02 | mirror | 5.28 | Solitary |
| 2020 | 1 | C10 | 270 | 21 | 544.56 | 3232.84 | 5.19 | 76.38 | mirror | 4.91 | Solitary |
| 2020 | 2 | C10 | 0 | 22 | 506.26 | 2292.82 | 3.83 | 47.95 | mirror | 4.91 | Solitary |
| 2020 | 3 | C10 | 300 | 23 | 573.66 | 3412.5 | 7.73 | 76.58 | mirror | 4.91 | Solitary |
| 2020 | 1 | C11 | 0 | 24 | 417.22 | 3378.99 | 5.02 | 84.15 | mirror | 6.39 | Solitary |
| 2020 | 2 | C11 | 0 | 25 | 475.56 | 1358.67 | 4.31 | 116.81 | mirror | 6.39 | Solitary |
| 2020 | 3 | C11 | 0 | 26 | 496.63 | 2351.44 | 4.1 | 59.99 | mirror | 6.39 | Solitary |
| 2020 | 1 | C12 | 140 | 27 | 477.17 | 3466.44 | 2.83 | 73.74 | mirror | 5.97 | Solitary |
| 2020 | 2 | C12 | 0 | 28 | 477.86 | 1764.33 | 3.03 | 49.33 | mirror | 5.97 | Solitary |
| 2020 | 3 | C12 | 300 | 29 | 492.53 | 65.34 | 0.11 | 2 | mirror | 5.97 | Solitary |
| 2020 | 1 | C13 | 0 | 30 | 383.83 | 1514.36 | 2.09 | 34.65 | mirror | 8.23 | Solitary |
| 2020 | 2 | C13 | 0 | 31 | 458.98 | 1613.67 | 3.33 | 73.33 | mirror | 8.23 | Solitary |
| 2020 | 3 | C13 | 300 | 19 | 405.24 | 20.85 | 0.04 | 0.9 | mirror | 8.23 | Solitary |
| 2020 | 1 | C14 | 0 | 20 | 455.61 | 2508.73 | 4.11 | 91.84 | mirror | 5.99 | Solitary |
| 2020 | 2 | C14 | 0 | 21 | 500.22 | 1574.57 | 2.65 | 55.52 | mirror | 5.99 | Solitary |
| 2020 | 3 | C14 | 300 | 22 | 556.92 | 115.42 | 0.23 | 4.07 | mirror | 5.99 | Solitary |
| 2020 | 1 | C15 | 0 | 23 | 511.17 | 4926.62 | 3.94 | 105.46 | mirror | 6.43 | Solitary |
| 2020 | 2 | C15 | 0 | 24 | 516.48 | 2017.18 | 3.34 | 55.17 | mirror | 6.43 | Solitary |
| 2020 | 3 | C15 | 300 | 25 | 400.28 | 19.02 | 0.03 | 0.59 | mirror | 6.43 | Solitary |
| 2020 | 1 | C16 | 0 | 26 | 554.78 | 4258.49 | 7.16 | 83.55 | mirror | 8.17 | Solitary |
| 2020 | 2 | C16 | 300 | 27 | 546.15 | 3194.1 | 6.65 | 85.97 | mirror | 8.17 | Solitary |
| 2020 | 3 | C16 | 0 | 28 | 528.34 | 64.45 | 0.11 | 2.04 | mirror | 8.17 | Solitary |
| 2020 | 1 | C17 | 0 | 29 | 541.5 | 6479.15 | 9.48 | 160.73 | mirror | 5.14 | Solitary |
| 2020 | 2 | C17 | 0 | 30 | 531.01 | 2316.96 | 4.22 | 58.48 | mirror | 5.14 | Solitary |
| 2020 | 3 | C17 | 0 | 31 | 496.17 | 66.77 | 0.15 | 1.63 | mirror | 5.14 | Solitary |
| 2020 | 1 | C2 | 300 | 19 | 423.15 | 1774.4 | 1.48 | 37.31 | mirror | 6.4 | Solitary |
| 2020 | 2 | C2 | 300 | 20 | 495.37 | 2647.91 | 2.2 | 59.16 | mirror | 6.4 | Solitary |
| 2020 | 3 | C2 | 300 | 21 | 511.28 | 1929.91 | 3.21 | 40.16 | mirror | 6.4 | Solitary |
| 2020 | 1 | C3 | 300 | 22 | 341.09 | 1152.4 | 1.91 | 34.66 | mirror | 5.56 | Solitary |
| 2020 | 2 | C3 | 0 | 23 | 474.95 | 1428.7 | 1.19 | 25.83 | mirror | 5.56 | Solitary |
| 2020 | 3 | C3 | 0 | 24 | 384.75 | 631.45 | 0.53 | 12.68 | mirror | 5.56 | Solitary |
| 2020 | 1 | C4 | 0 | 25 | 375.47 | 2279.46 | 3.77 | 56.85 | mirror | 6.37 | Solitary |
| 2020 | 2 | C4 | 0 | 26 | 536.94 | 2736.4 | 4.69 | 77.34 | mirror | 6.37 | Solitary |
| 2020 | 3 | C4 | 0 | 27 | 499.89 | 1608.64 | 2.45 | 45.12 | mirror | 6.37 | Solitary |
| 2020 | 1 | C5 | 120 | 28 | 489.91 | 1924.64 | 4.43 | 70.4 | mirror | 10.45 | Solitary |
| 2020 | 2 | C5 | 300 | 29 | 507.11 | 2045.39 | 3.23 | 55.13 | mirror | 10.45 | Solitary |
| 2020 | 3 | C5 | 300 | 30 | 464.88 | 4001.64 | 8.17 | 105.46 | mirror | 10.45 | Solitary |
| 2020 | 1 | C6 | 300 | 31 | 380.87 | 1067.69 | 1.81 | 37.49 | mirror | 10.01 | Solitary |
| 2020 | 2 | C6 | 0 | 19 | 476.08 | 1350.62 | 4.35 | 99.27 | mirror | 10.01 | Solitary |
| 2020 | 3 | C6 | 0 | 20 | 519.41 | 1691.18 | 2.63 | 42.18 | mirror | 10.01 | Solitary |
| 2020 | 1 | C8 | 300 | 21 | 559.46 | 3701.42 | 8.33 | 164.66 | mirror | 10.41 | Solitary |
| 2020 | 2 | C8 | 10 | 22 | 496.19 | 1679.43 | 3.46 | 71.87 | mirror | 10.41 | Solitary |
| 2020 | 3 | C8 | 0 | 23 | 435.23 | 27.26 | 0.03 | 0.58 | mirror | 10.41 | Solitary |
| 2020 | 1 | C9 | 0 | 24 | 435.87 | 1222.46 | 1.58 | 42.4 | mirror | 10.65 | Solitary |
| 2020 | 2 | C9 | 0 | 25 | 513.9 | 2869.98 | 4.89 | 87.46 | mirror | 10.65 | Solitary |
| 2020 | 3 | C9 | 300 | 26 | 507.82 | 1721.35 | 2.92 | 47.9 | mirror | 10.65 | Solitary |
| 2020 | 1 | S1 | 0 | 27 | 517.17 | 2713.6 | 4.56 | 46.8 | mirror | 6.28 | Solitary |
| 2020 | 2 | S1 | 300 | 28 | 547.95 | 6381.05 | 11.2 | 150.76 | mirror | 6.28 | Solitary |
| 2020 | 3 | S1 | 300 | 29 | 547.31 | 236.52 | 0.4 | 4.33 | mirror | 6.28 | Solitary |
| 2020 | 1 | S10 | 300 | 30 | 521.61 | 1844.7 | 4.68 | 85.55 | mirror | 6.86 | Solitary |
| 2020 | 2 | S10 | 0 | 31 | 517.9 | 1891.28 | 1.58 | 26.97 | mirror | 6.86 | Solitary |
| 2020 | 3 | S10 | 0 | 19 | 306.55 | 1726.48 | 6.73 | 103.42 | mirror | 6.86 | Solitary |
| 2020 | 1 | S11 | 300 | 20 | 435.28 | 1337.33 | 2.17 | 27.52 | mirror | 7.14 | Solitary |
| 2020 | 2 | S11 | 0 | 21 | 530.27 | 3015.43 | 5.02 | 100.17 | mirror | 7.14 | Solitary |
| 2020 | 3 | S11 | 0 | 22 | 543.69 | 3011.54 | 5.91 | 94.73 | mirror | 7.14 | Solitary |
| 2020 | 1 | S12 | 0 | 23 | 500.61 | 1998.69 | 1.6 | 32.4 | mirror | 7.73 | Solitary |
| 2020 | 2 | S12 | 0 | 24 | 501.97 | 1760.77 | 4.09 | 69.18 | mirror | 7.73 | Solitary |
| 2020 | 3 | S12 | 0 | 25 | 573.42 | 8904.5 | 10.55 | 302.59 | mirror | 7.73 | Solitary |
| 2020 | 1 | S13 | 300 | 26 | 530.56 | 3435.99 | 6.47 | 62.46 | mirror | 6.91 | Solitary |
| 2020 | 2 | S13 | 300 | 27 | 495.04 | 4264.88 | 7.25 | 103.12 | mirror | 6.91 | Solitary |
| 2020 | 3 | S13 | 0 | 28 | 548.59 | 2549.26 | 5.61 | 99.26 | mirror | 6.91 | Solitary |
| 2020 | 1 | S14 | 0 | 29 | 573.33 | 5474.19 | 8.62 | 107.01 | mirror | 7.74 | Solitary |
| 2020 | 2 | S14 | 0 | 30 | 590.93 | 4440.26 | 7.12 | 106.13 | mirror | 7.74 | Solitary |
| 2020 | 3 | S14 | 0 | 31 | 544.58 | 3890.12 | 6.61 | 66.24 | mirror | 7.74 | Solitary |
| 2020 | 1 | S16 | 0 | 19 | 431.33 | 938.45 | 0.75 | 13.39 | mirror | 7.25 | Solitary |
| 2020 | 2 | S16 | 0 | 20 | 538.38 | 2199.5 | 7.08 | 83.86 | mirror | 7.25 | Solitary |
| 2020 | 3 | S16 | 0 | 21 | 499.3 | 3660.69 | 8.08 | 162.27 | mirror | 7.25 | Solitary |
| 2020 | 1 | S17 | 0 | 22 | 503 | 1825.06 | 2.61 | 43.81 | mirror | 8.95 | Solitary |
| 2020 | 2 | S17 | 0 | 23 | 506.52 | 1976.2 | 5.77 | 125.9 | mirror | 8.95 | Solitary |
| 2020 | 3 | S17 | 0 | 24 | 590.22 | 5706.6 | 9.52 | 116.46 | mirror | 8.95 | Solitary |
| 2020 | 1 | S2 | 0 | 25 | 467.22 | 2813.15 | 5.12 | 55.28 | mirror | 6.27 | Solitary |
| 2020 | 2 | S2 | 300 | 26 | 547.43 | 3318.46 | 5.6 | 79.24 | mirror | 6.27 | Solitary |
| 2020 | 3 | S2 | 0 | 27 | 533.9 | 179.38 | 0.31 | 3.5 | mirror | 6.27 | Solitary |
| 2020 | 1 | S3 | 0 | 28 | 289.33 | 711.91 | 2.05 | 39.29 | mirror | 7.91 | Solitary |
| 2020 | 2 | S3 | 300 | 29 | 542.61 | 3282.96 | 6.71 | 106.11 | mirror | 7.91 | Solitary |
| 2020 | 3 | S3 | 300 | 30 | 516.44 | 105.99 | 0.2 | 4.72 | mirror | 7.91 | Solitary |
| 2020 | 1 | S4 | 300 | 31 | 274.22 | 522.75 | 1 | 18.78 | mirror | 6.72 | Solitary |
| 2020 | 2 | S4 | 300 | 19 | 573.77 | 2896.3 | 3.64 | 62.22 | mirror | 6.72 | Solitary |
| 2020 | 3 | S4 | 0 | 20 | 520.05 | 64.94 | 0.23 | 3.61 | mirror | 6.72 | Solitary |
| 2020 | 1 | S5 | 300 | 21 | 506.5 | 3454.98 | 5.73 | 95.13 | mirror | 7.59 | Solitary |
| 2020 | 2 | S5 | 0 | 22 | 568.57 | 4062.42 | 6.74 | 93.53 | mirror | 7.59 | Solitary |
| 2020 | 3 | S5 | 300 | 23 | 544.22 | 3106.55 | 5.32 | 104.54 | mirror | 7.59 | Solitary |
| 2020 | 1 | S6 | 276 | 24 | 547.44 | 5023.3 | 4.11 | 92.7 | mirror | 7.34 | Solitary |
| 2020 | 2 | S6 | 0 | 25 | 561.85 | 4366.97 | 9.57 | 141.91 | mirror | 7.34 | Solitary |
| 2020 | 3 | S6 | 0 | 26 | 510.7 | 57.96 | 0.14 | 1.94 | mirror | 7.34 | Solitary |
| 2020 | 1 | S7 | 0 | 27 | 431.67 | 1537.65 | 2.27 | 44.47 | mirror | 6.82 | Solitary |
| 2020 | 2 | S7 | 0 | 28 | 522.56 | 2705.63 | 4.64 | 73.83 | mirror | 6.82 | Solitary |
| 2020 | 3 | S7 | 0 | 29 | 524.5 | 12831.61 | 17.83 | 557.84 | mirror | 6.82 | Solitary |
| 2020 | 1 | S8 | 0 | 30 | 504.5 | 1742.78 | 1.42 | 32.68 | mirror | 7.94 | Solitary |
| 2020 | 2 | S8 | 0 | 31 | 561.8 | 4471.19 | 8.4 | 116.1 | mirror | 7.94 | Solitary |
| 2020 | 3 | S8 | 0 | 19 | 504.47 | 2042.36 | 3.25 | 69.05 | mirror | 7.94 | Solitary |
| 2020 | 1 | S9 | 300 | 20 | 513.56 | 3454.98 | 2.81 | 70.75 | mirror | 7.01 | Solitary |
| 2020 | 2 | S9 | 46 | 21 | 477.07 | 1199.27 | 1 | 22.08 | mirror | 7.01 | Solitary |
| 2020 | 3 | S9 | 300 | 22 | 338.75 | 1396.11 | 2.09 | 35.35 | mirror | 7.01 | Solitary |
| 2021 | 1 | C18 | 300 | 23 | 720.72 | 4962.35 | 10.25 | 82.03 | open-field | 4.92 | Solitary |
| 2021 | 2 | C18 | 300 | 24 | 693.94 | 4128.51 | 10.92 | 52.48 | open-field | 4.92 | Solitary |
| 2021 | 3 | C18 | 300 | 25 | 693.17 | 3922.98 | 21.55 | 48.61 | open-field | 4.92 | Solitary |
| 2021 | 1 | C19 | 300 | 26 | 720.72 | 4856.89 | 13.5 | 96.81 | open-field | 5.47 | Solitary |
| 2021 | 2 | C19 | 80 | 27 | 617.56 | 1984.6 | 5.88 | 36.02 | open-field | 5.47 | Solitary |
| 2021 | 3 | C19 | 300 | 28 | 686 | 3329.05 | 11.9 | 47.76 | open-field | 5.47 | Solitary |
| 2021 | 1 | C20 | 300 | 29 | 725.67 | 5195.29 | 7.29 | 91.01 | open-field | 4.81 | Solitary |
| 2021 | 2 | C20 | 0 | 30 | 671.06 | 2580.16 | 4.18 | 39.94 | open-field | 4.81 | Solitary |
| 2021 | 3 | C20 | 300 | 31 | 744.11 | 3707.39 | 5.37 | 43.91 | open-field | 4.81 | Solitary |
| 2021 | 1 | C21 | 300 | 106 | 766.33 | 6741.19 | 15.52 | 135.91 | open-field | 5.01 | Solitary |
| 2021 | 2 | C21 | 300 | 98 | 685.67 | 5178.81 | 6.53 | 59.21 | open-field | 5.01 | Solitary |
| 2021 | 3 | C21 | 300 | 92 | 726.22 | 5195.45 | 8.94 | 59.27 | open-field | 5.01 | Solitary |
| 2021 | 1 | C22 | 300 | 54 | 605 | 3072.71 | 20.16 | 62.3 | open-field | 5.09 | Solitary |
| 2021 | 2 | C22 | 300 | 41 | 616.83 | 2351.19 | 8.3 | 40.37 | open-field | 5.09 | Solitary |
| 2021 | 3 | C22 | 300 | 52 | 688.56 | 2859.51 | 9.65 | 42.29 | open-field | 5.09 | Solitary |
| 2021 | 1 | C23 | 290 | 127 | 648.61 | 4962.26 | 32.99 | 72.89 | open-field | 5.66 | Solitary |
| 2021 | 2 | C23 | 300 | 77 | 643.22 | 2528.62 | 25.83 | 37.46 | open-field | 5.66 | Solitary |
| 2021 | 3 | C23 | 300 | 51 | 707.67 | 2552.32 | 21.02 | 43.3 | open-field | 5.66 | Solitary |
| 2021 | 1 | C24 | 300 | 55 | 648.61 | 2959.54 | 6.9 | 46.03 | open-field | 5.18 | Solitary |
| 2021 | 2 | C24 | 300 | 88 | 742.72 | 4752.58 | 9.58 | 57.94 | open-field | 5.18 | Solitary |
| 2021 | 3 | C24 | 300 | 72 | 685.17 | 4486.95 | 4.39 | 44.22 | open-field | 5.18 | Solitary |
| 2021 | 1 | C25 | 255 | 105 | 738.61 | 5285.49 | 12.27 | 102.67 | open-field | 4.85 | Solitary |
| 2021 | 2 | C25 | 300 | 43 | 688.22 | 3383.92 | 5.99 | 40.36 | open-field | 4.85 | Solitary |
| 2021 | 1 | C26 | 300 | 79 | 609.94 | 3072.01 | 17.12 | 48.66 | open-field | 5.04 | Solitary |
| 2021 | 2 | C26 | 300 | 64 | 642.83 | 2947.64 | 28.81 | 43.33 | open-field | 5.04 | Solitary |
| 2021 | 3 | C26 | 300 | 71 | 718 | 3746.84 | 26.68 | 53.24 | open-field | 5.04 | Solitary |
| 2021 | 2 | C27 | 0 | 50 | 607.39 | 2892.37 | 4.06 | 65.64 | open-field | 5.37 | Solitary |
| 2021 | 3 | C27 | 300 | 47 | 646.5 | 3266.61 | 3.88 | 51.39 | open-field | 5.37 | Solitary |
| 2021 | 3 | C27 | 270 | 41 | 790.94 | 3398.42 | 6.7 | 48.32 | open-field | 5.37 | Solitary |
| 2021 | 1 | C28 | 40 | 104 | 757.83 | 6140.07 | 10.95 | 59.14 | open-field | 5.71 | Solitary |
| 2021 | 2 | C28 | 300 | 77 | 688.72 | 4771.75 | 8.16 | 61.92 | open-field | 5.71 | Solitary |
| 2021 | 3 | C28 | 300 | 85 | 733.94 | 5204.8 | 17.54 | 64.01 | open-field | 5.71 | Solitary |
| 2021 | 1 | C29 | 90 | 102 | 736.28 | 5635.02 | 10.51 | 54.02 | open-field | 5.17 | Solitary |
| 2021 | 2 | C29 | 0 | 83 | 570.28 | 3063.56 | 14.92 | 46.34 | open-field | 5.17 | Solitary |
| 2021 | 3 | C29 | 300 | 30 | 422.28 | 2095.9 | 6.67 | 51.02 | open-field | 5.17 | Solitary |
| 2021 | 1 | C30 | 135 | 20 | 462.22 | 1040.32 | 1.97 | 25.36 | open-field | 5.85 | Solitary |
| 2021 | 2 | C30 | 300 | 37 | 714.06 | 2173.63 | 4.31 | 41.91 | open-field | 5.85 | Solitary |
| 2021 | 3 | C30 | 300 | 54 | 655.56 | 2484.74 | 26.77 | 40.91 | open-field | 5.85 | Solitary |
| 2021 | 2 | C31 | 300 | 43 | 416.33 | 889.65 | 1.32 | 19.86 | open-field | 4.72 | Solitary |
| 2021 | 3 | C31 | 300 | 52 | 664.17 | 3083.48 | 4.06 | 43.38 | open-field | 4.72 | Solitary |
| 2021 | 3 | C31 | 300 | 61 | 620.39 | 2649.59 | 14.12 | 48.05 | open-field | 4.72 | Solitary |
| 2021 | 1 | C32 | 300 | 131 | 735.83 | 4683.42 | 26.84 | 51.47 | open-field | 5.4 | Solitary |
| 2021 | 2 | C32 | 300 | 83 | 712.67 | 4047.84 | 17.85 | 52.91 | open-field | 5.4 | Solitary |
| 2021 | 3 | C32 | 300 | 68 | 754.44 | 3969.85 | 37.37 | 50.76 | open-field | 5.4 | Solitary |
| 2021 | 1 | C33 | 0 | 130 | 704.28 | 5756.42 | 17.08 | 74.19 | open-field | 5.37 | Solitary |
| 2021 | 2 | C33 | 300 | 181 | 662.78 | 6047.24 | 17.47 | 65.48 | open-field | 5.37 | Solitary |
| 2021 | 3 | C33 | 300 | 75 | 745.67 | 4433.21 | 57.84 | 50.96 | open-field | 5.37 | Solitary |
| 2021 | 2 | C34 | 0 | 136 | 808 | 7428.26 | 12.59 | 243.09 | open-field | 4.36 | Solitary |
| 2021 | 3 | C34 | 300 | 95 | 540.61 | 2877.69 | 14.2 | 37.97 | open-field | 4.36 | Solitary |
| 2021 | 3 | C34 | 225 | 76 | 753.78 | 5567.03 | 24.43 | 70.83 | open-field | 4.36 | Solitary |
| 2021 | 2 | C35 | 300 | 65 | 637.17 | 2263.98 | 26.65 | 35.55 | open-field | 4.8 | Solitary |
| 2021 | 3 | C35 | 300 | 36 | 492.39 | 1456.75 | 20.62 | 23.86 | open-field | 4.8 | Solitary |
| 2021 | 2 | C36 | 75 | 62 | 754.5 | 4418.01 | 10.56 | 75.09 | open-field | 5.58 | Solitary |
| 2021 | 3 | C36 | 300 | 95 | 721.06 | 5294.82 | 7.2 | 63.24 | open-field | 5.58 | Solitary |
| 2021 | 3 | C36 | 300 | 77 | 780 | 5004.45 | 6.78 | 64.42 | open-field | 5.58 | Solitary |
| 2021 | 1 | C37 | 300 | 140 | 746.22 | 6241.04 | 18.16 | 66.17 | open-field | 5.46 | Solitary |
| 2021 | 2 | C37 | 300 | 99 | 663 | 4849.08 | 9.21 | 55.5 | open-field | 5.46 | Solitary |
| 2021 | 3 | C37 | 300 | 77 | 759.5 | 5037.83 | 6.96 | 59.12 | open-field | 5.46 | Solitary |
| 2021 | 2 | C38 | 300 | 88 | 681.67 | 3858.37 | 11.98 | 56.96 | open-field | 4.56 | Solitary |
| 2021 | 3 | C38 | 300 | 57 | 697.61 | 3106.13 | 8.16 | 40.55 | open-field | 4.56 | Solitary |
| 2021 | 3 | C38 | 0 | 56 | 714.5 | 4168.38 | 5.99 | 59.09 | open-field | 4.56 | Solitary |
| 2021 | 1 | C39 | 300 | 128 | 681.78 | 6664.36 | 21.98 | 104.91 | open-field | 4.62 | Solitary |
| 2021 | 2 | C39 | 210 | 105 | 651.83 | 5399.88 | 8.45 | 73.22 | open-field | 4.62 | Solitary |
| 2021 | 3 | C39 | 300 | 111 | 721.56 | 5955.24 | 12.75 | 66.47 | open-field | 4.62 | Solitary |
| 2021 | 1 | C40 | 300 | 64 | 675.33 | 3307.97 | 7.38 | 44.81 | open-field | 5.2 | Solitary |
| 2021 | 2 | C40 | 300 | 76 | 685.5 | 3641.8 | 13.99 | 46.21 | open-field | 5.2 | Solitary |
| 2021 | 3 | C40 | 300 | 74 | 758.72 | 4776.95 | 30.86 | 59.99 | open-field | 5.2 | Solitary |
| 2021 | 1 | C41 | 300 | 48 | 711.44 | 3168.22 | 12.78 | 40.33 | open-field | 5.3 | Solitary |
| 2021 | 2 | C41 | 50 | 50 | 711.11 | 2720.14 | 6.48 | 41.38 | open-field | 5.3 | Solitary |
| 2021 | 3 | C41 | 300 | 83 | 721.44 | 4048.45 | 14.48 | 47.68 | open-field | 5.3 | Solitary |
| 2021 | 2 | C42 | 300 | 64 | 705.44 | 3441 | 12.51 | 45.39 | open-field | 6 | Solitary |
| 2021 | 3 | C42 | 300 | 36 | 673.17 | 1980.56 | 1.97 | 34.82 | open-field | 6 | Solitary |
| 2021 | 3 | C42 | 300 | 42 | 643.83 | 2908.34 | 5.03 | 50.97 | open-field | 6 | Solitary |
| 2021 | 1 | C44 | 300 | 45 | 796.5 | 3339.7 | 6.36 | 107.49 | open-field | 4.76 | Solitary |
| 2021 | 2 | C44 | 300 | 31 | 732.17 | 2886.15 | 2.74 | 30.15 | open-field | 4.76 | Solitary |
| 2021 | 3 | C44 | 300 | 51 | 650.83 | 2374.22 | 5.09 | 41.27 | open-field | 4.76 | Solitary |
| 2021 | 2 | C45 | 0 | 75 | 731.89 | 4756.81 | 9.87 | 65.93 | open-field | 4.74 | Solitary |
| 2021 | 3 | C45 | 300 | 54 | 719.83 | 3378.65 | 7.12 | 46.35 | open-field | 4.74 | Solitary |
| 2021 | 3 | C45 | 85 | 61 | 620.5 | 3680.8 | 4.65 | 139.46 | open-field | 4.74 | Solitary |
| 2021 | 2 | C46 | 300 | 56 | 607.72 | 1661.03 | 14 | 35.43 | open-field | 4.54 | Solitary |
| 2021 | 3 | C46 | 300 | 74 | 687.33 | 3574.22 | 15.48 | 44.67 | open-field | 4.54 | Solitary |
| 2021 | 3 | C46 | 300 | 66 | 673.94 | 3830.97 | 7.36 | 57.13 | open-field | 4.54 | Solitary |
| 2021 | 1 | C47 | 0 | 95 | 698.39 | 4756.19 | 23 | 107.09 | open-field | 5.18 | Solitary |
| 2021 | 2 | C47 | 300 | 79 | 684.72 | 3982.46 | 8.43 | 49.81 | open-field | 5.18 | Solitary |
| 2021 | 3 | C47 | 300 | 63 | 669.94 | 3472.15 | 5.98 | 53.58 | open-field | 5.18 | Solitary |
| 2021 | 1 | C18 | 0 | 10 | 434.5 | 1427.34 | 2.64 | 33.44 | mirror | 4.92 | Solitary |
| 2021 | 2 | C18 | 105 | 28 | 557.11 | 2292.44 | 3.65 | 38.61 | mirror | 4.92 | Solitary |
| 2021 | 3 | C18 | 300 | 28 | 551.11 | 2631.5 | 4.23 | 48.81 | mirror | 4.92 | Solitary |
| 2021 | 1 | C19 | 300 | 17 | 445.83 | 1268.43 | 2.41 | 28.92 | mirror | 5.47 | Solitary |
| 2021 | 2 | C19 | 300 | 16 | 489.61 | 1761.43 | 2.79 | 27.96 | mirror | 5.47 | Solitary |
| 2021 | 3 | C19 | 0 | 10 | 430.28 | 883.13 | 1.9 | 30.4 | mirror | 5.47 | Solitary |
| 2021 | 1 | C20 | 300 | 7 | 368.61 | 1999.75 | 4.73 | 91.95 | mirror | 4.81 | Solitary |
| 2021 | 2 | C20 | 300 | 6 | 527.22 | 1603.9 | 2.41 | 36.24 | mirror | 4.81 | Solitary |
| 2021 | 3 | C20 | 300 | 13 | 504.17 | 1853.7 | 2.87 | 52.27 | mirror | 4.81 | Solitary |
| 2021 | 1 | C21 | 300 | 3 | 441.72 | 2765.44 | 5.76 | 94.86 | mirror | 5.01 | Solitary |
| 2021 | 2 | C21 | 300 | 4 | 555.56 | 2669.32 | 4.83 | 47.1 | mirror | 5.01 | Solitary |
| 2021 | 3 | C21 | 300 | 5 | 507.94 | 1709.32 | 3.26 | 41.96 | mirror | 5.01 | Solitary |
| 2021 | 1 | C22 | 300 | 4 | 357.17 | 1453.55 | 2.44 | 50.3 | mirror | 5.09 | Solitary |
| 2021 | 2 | C22 | 300 | 6 | 493.83 | 1563.83 | 2.46 | 33.27 | mirror | 5.09 | Solitary |
| 2021 | 3 | C22 | 300 | 5 | 497.06 | 1596.69 | 3.76 | 43.25 | mirror | 5.09 | Solitary |
| 2021 | 1 | C23 | 300 | 12 | 439.83 | 1304.19 | 2.29 | 36.56 | mirror | 5.66 | Solitary |
| 2021 | 2 | C23 | 50 | 7 | 420.89 | 996.94 | 1.54 | 28.21 | mirror | 5.66 | Solitary |
| 2021 | 3 | C23 | 300 | 25 | 596.56 | 2819.45 | 4.36 | 59.35 | mirror | 5.66 | Solitary |
| 2021 | 1 | C24 | 15 | 9 | 451.06 | 1377.62 | 3.13 | 40.88 | mirror | 5.18 | Solitary |
| 2021 | 2 | C24 | 300 | 8 | 554.11 | 3005.85 | 4.6 | 42.68 | mirror | 5.18 | Solitary |
| 2021 | 3 | C24 | 280 | 29 | 553.5 | 2826.66 | 4.6 | 51.11 | mirror | 5.18 | Solitary |
| 2021 | 1 | C25 | 60 | 2 | 510.17 | 3223.58 | 6.11 | 176.39 | mirror | 4.85 | Solitary |
| 2021 | 2 | C25 | 45 | 17 | 550.61 | 2489.19 | 3.91 | 44.15 | mirror | 4.85 | Solitary |
| 2021 | 3 | C25 | 300 | 11 | 517.5 | 2029.61 | 5.97 | 46.1 | mirror | 4.85 | Solitary |
| 2021 | 1 | C26 | 300 | 17 | 484.83 | 2842.48 | 5.76 | 151.99 | mirror | 5.04 | Solitary |
| 2021 | 2 | C26 | 300 | 11 | 504.33 | 1821.63 | 3.04 | 34.2 | mirror | 5.04 | Solitary |
| 2021 | 3 | C26 | 300 | 13 | 489.11 | 1687.65 | 2.66 | 43.86 | mirror | 5.04 | Solitary |
| 2021 | 1 | C27 | 60 | 5 | 506.11 | 3403.04 | 5.29 | 121.38 | mirror | 5.37 | Solitary |
| 2021 | 2 | C27 | 300 | 10 | 510.67 | 2035.4 | 4.04 | 40.85 | mirror | 5.37 | Solitary |
| 2021 | 3 | C27 | 0 | 7 | 533.83 | 2266.15 | 3.5 | 45.61 | mirror | 5.37 | Solitary |
| 2021 | 1 | C28 | 30 | 3 | 476.22 | 2939.02 | 7.61 | 194.89 | mirror | 5.71 | Solitary |
| 2021 | 2 | C28 | 300 | 23 | 562.39 | 2916.01 | 4.62 | 49.93 | mirror | 5.71 | Solitary |
| 2021 | 3 | C28 | 300 | 31 | 592.33 | 2576.77 | 4.08 | 53.11 | mirror | 5.71 | Solitary |
| 2021 | 1 | C29 | 300 | 7 | 164.83 | 986.03 | 3.59 | 58.98 | mirror | 5.17 | Solitary |
| 2021 | 2 | C29 | 300 | 15 | 541.5 | 3441.61 | 5.64 | 96.74 | mirror | 5.17 | Solitary |
| 2021 | 3 | C29 | 300 | 13 | 526.17 | 2439.96 | 3.8 | 50.12 | mirror | 5.17 | Solitary |
| 2021 | 1 | C30 | 300 | 11 | 495.22 | 1723.18 | 2.94 | 42.18 | mirror | 5.85 | Solitary |
| 2021 | 2 | C30 | 300 | 6 | 484.89 | 1202.34 | 1.79 | 32.16 | mirror | 5.85 | Solitary |
| 2021 | 3 | C30 | 300 | 6 | 503.11 | 1653.62 | 2.65 | 45.65 | mirror | 5.85 | Solitary |
| 2021 | 1 | C31 | 300 | 2 | 338.94 | 2291.18 | 8.92 | 75.81 | mirror | 4.72 | Solitary |
| 2021 | 2 | C31 | 300 | 3 | 366.61 | 819.01 | 2.69 | 31.72 | mirror | 4.72 | Solitary |
| 2021 | 3 | C31 | 300 | 5 | 462.06 | 1572.29 | 2.35 | 34.55 | mirror | 4.72 | Solitary |
| 2021 | 1 | C32 | 45 | 1 | 527 | 2511.53 | 2.03 | 33.06 | mirror | 5.4 | Solitary |
| 2021 | 2 | C32 | 300 | 17 | 565 | 2580.87 | 4.08 | 46.87 | mirror | 5.4 | Solitary |
| 2021 | 3 | C32 | 300 | 10 | 526.11 | 2078.52 | 3.6 | 42.3 | mirror | 5.4 | Solitary |
| 2021 | 1 | C33 | 0 | 9 | 463.44 | 2488.02 | 6.06 | 72.33 | mirror | 5.37 | Solitary |
| 2021 | 2 | C33 | 0 | 26 | 536.5 | 3010.37 | 5.89 | 42.61 | mirror | 5.37 | Solitary |
| 2021 | 3 | C33 | 0 | 13 | 397.83 | 1507.12 | 4.61 | 37.82 | mirror | 5.37 | Solitary |
| 2021 | 1 | C34 | 20 | 4 | 50.78 | 432.85 | 6.72 | 63.24 | mirror | 4.36 | Solitary |
| 2021 | 2 | C34 | 30 | 45 | 522 | 3132.36 | 5.05 | 38.95 | mirror | 4.36 | Solitary |
| 2021 | 3 | C34 | 90 | 23 | 529.44 | 3829.45 | 6.07 | 97.58 | mirror | 4.36 | Solitary |
| 2021 | 1 | C35 | 0 | 22 | 447.39 | 1380.56 | 2.7 | 29.84 | mirror | 4.8 | Solitary |
| 2021 | 2 | C35 | 300 | 17 | 403.22 | 1054.33 | 1.96 | 23.2 | mirror | 4.8 | Solitary |
| 2021 | 1 | C36 | 225 | 2 | 543.06 | 2554.31 | 5.78 | 76.51 | mirror | 5.58 | Solitary |
| 2021 | 2 | C36 | 300 | 16 | 553.44 | 2534.32 | 4.11 | 51.81 | mirror | 5.58 | Solitary |
| 2021 | 3 | C36 | 300 | 28 | 587.56 | 3189.13 | 4.98 | 47.95 | mirror | 5.58 | Solitary |
| 2021 | 1 | C37 | 210 | 5 | 555.83 | 4975.2 | 9.76 | 255.19 | mirror | 5.46 | Solitary |
| 2021 | 2 | C37 | 220 | 18 | 522.11 | 2895.15 | 8.36 | 53.92 | mirror | 5.46 | Solitary |
| 2021 | 3 | C37 | 0 | 14 | 545.06 | 2523.86 | 4.42 | 50.82 | mirror | 5.46 | Solitary |
| 2021 | 1 | C38 | 5 | 12 | 504.39 | 3136.75 | 7.79 | 160.02 | mirror | 4.56 | Solitary |
| 2021 | 2 | C38 | 300 | 23 | 540.11 | 2619.6 | 4.24 | 43.08 | mirror | 4.56 | Solitary |
| 2021 | 3 | C38 | 300 | 13 | 554.17 | 4050.33 | 6.46 | 97.42 | mirror | 4.56 | Solitary |
| 2021 | 1 | C39 | 0 | 1 | 71.17 | 274.16 | 0.22 | 5.36 | mirror | 4.62 | Solitary |
| 2021 | 2 | C39 | 145 | 7 | 482.22 | 1732.96 | 3.72 | 39.22 | mirror | 4.62 | Solitary |
| 2021 | 3 | C39 | 300 | 10 | 494.44 | 1867.7 | 3.78 | 34.4 | mirror | 4.62 | Solitary |
| 2021 | 1 | C40 | 300 | 28 | 502.5 | 1309.31 | 2.02 | 30.1 | mirror | 5.2 | Solitary |
| 2021 | 2 | C40 | 300 | 16 | 524.72 | 1626.89 | 2.76 | 31.57 | mirror | 5.2 | Solitary |
| 2021 | 3 | C40 | 300 | 38 | 577.44 | 3641.2 | 5.83 | 50.91 | mirror | 5.2 | Solitary |
| 2021 | 1 | C41 | 0 | 43 | 580 | 3939.72 | 6.35 | 54.53 | mirror | 5.3 | Solitary |
| 2021 | 2 | C41 | 0 | 29 | 548.94 | 2581.05 | 4.18 | 37.91 | mirror | 5.3 | Solitary |
| 2021 | 3 | C41 | 300 | 14 | 565.61 | 3145.94 | 5.69 | 55.39 | mirror | 5.3 | Solitary |
| 2021 | 1 | C42 | 0 | 9 | 498.44 | 1980.65 | 3.04 | 34.86 | mirror | 6 | Solitary |
| 2021 | 2 | C42 | 300 | 1 | 509.44 | 2320.87 | 1.88 | 29.27 | mirror | 6 | Solitary |
| 2021 | 3 | C42 | 300 | 27 | 556.11 | 2635.67 | 4.34 | 42.33 | mirror | 6 | Solitary |
| 2021 | 1 | C44 | 300 | 4 | 467.89 | 1542.55 | 3.53 | 61.63 | mirror | 4.76 | Solitary |
| 2021 | 2 | C44 | 300 | 5 | 492.94 | 1478.23 | 4.04 | 35.63 | mirror | 4.76 | Solitary |
| 2021 | 3 | C44 | 35 | 26 | 565.56 | 2395.3 | 4 | 42.31 | mirror | 4.76 | Solitary |
| 2021 | 1 | C45 | 300 | 1 | 425.11 | 3455.85 | 2.84 | 66.15 | mirror | 4.74 | Solitary |
| 2021 | 3 | C45 | 20 | 29 | 560.89 | 3127.04 | 5.01 | 56.86 | mirror | 4.74 | Solitary |
| 2021 | 1 | C46 | 300 | 1 | 453.56 | 1282.65 | 1.05 | 18.32 | mirror | 4.54 | Solitary |
| 2021 | 2 | C46 | 300 | 4 | 504.22 | 1915.85 | 4.37 | 49.96 | mirror | 4.54 | Solitary |
| 2021 | 3 | C46 | 300 | 1 | 502.06 | 1625.94 | 1.31 | 17.31 | mirror | 4.54 | Solitary |
| 2021 | 1 | C47 | 0 | 1 | 455.22 | 1602.81 | 1.3 | 24.28 | mirror | 5.18 | Solitary |
| 2021 | 2 | C47 | 300 | 4 | 457.72 | 1312.78 | 2.03 | 27.62 | mirror | 5.18 | Solitary |
| 2021 | 3 | C47 | 300 | 6 | 544.61 | 2648.78 | 4.12 | 49.39 | mirror | 5.18 | Solitary |
| 2021 | 1 | C23 | 20 | 17 | 543.8333 | 2705.207 | 4.875224 | 65.12223 | mirror | 5.66 | Paired |
| 2021 | 2 | C23 | 300 | 10 | 549.2778 | 1832.506 | 2.769558 | 40.37545 | mirror | 5.66 | Paired |
| 2021 | 1 | C23 | 0 | 40 | 736 | 2987.444 | 3.494226 | 44.10951 | open-field | 5.66 | Paired |
| 2021 | 2 | C23 | 5 | 45 | 758.6667 | 2980.209 | 3.398499 | 46.3028 | open-field | 5.66 | Paired |
| 2021 | 3 | C23 | 300 | 24 | 583.1667 | 2146.015 | 3.146901 | 42.78954 | mirror | 5.66 | Paired |
| 2021 | 1 | C27 | 300 | 22 | 548.1667 | 2271.063 | 3.679649 | 49.45524 | mirror | 5.37 | Paired |
| 2021 | 2 | C27 | 300 | 18 | 504.3333 | 1925.241 | 3.476361 | 41.20332 | mirror | 5.37 | Paired |
| 2021 | 1 | C27 | 300 | 44 | 714.3333 | 3305.441 | 3.898734 | 50.87421 | open-field | 5.37 | Paired |
| 2021 | 2 | C27 | 300 | 62 | 761.7778 | 3859.848 | 4.273019 | 52.22253 | open-field | 5.37 | Paired |
| 2021 | 3 | C27 | 0 | 10 | 502.2222 | 1948.776 | 3.038635 | 43.60333 | mirror | 5.37 | Paired |
| 2021 | 1 | C30 | 300 | 13 | 489.7222 | 1851.802 | 3.333888 | 43.32784 | mirror | 5.85 | Paired |
| 2021 | 1 | C30 | 300 | 55 | 665.0556 | 3788.981 | 5.108246 | 62.28931 | open-field | 5.85 | Paired |
| 2021 | 2 | C30 | 20 | 13 | 552.1667 | 2923.237 | 4.606389 | 63.82867 | mirror | 5.85 | Paired |
| 2021 | 2 | C30 | 300 | 24 | 598.7222 | 3011.969 | 5.192444 | 67.15056 | open-field | 5.85 | Paired |
| 2021 | 3 | C30 | 300 | 5 | 547.3889 | 3442.432 | 6.281748 | 115.2816 | mirror | 5.85 | Paired |
| 2021 | 1 | C40 | 300 | 70 | 640.1667 | 2906.982 | 3.796519 | 44.46837 | open-field | 5.2 | Paired |
| 2021 | 2 | C40 | 300 | 60 | 668.2222 | 3034.346 | 4.122456 | 48.26612 | open-field | 5.2 | Paired |
| 2021 | 1 | C40 | 0 | 1 | 356.2778 | 679.1235 | 0.554185 | 12.97883 | mirror | 5.2 | Paired |
| 2021 | 2 | C40 | 300 | 11 | 390.1111 | 1131.925 | 2.758964 | 36.86681 | mirror | 5.2 | Paired |
| 2021 | 3 | C40 | 300 | 17 | 460.6667 | 2056.489 | 3.520759 | 45.73032 | mirror | 5.2 | Paired |
| 2021 | 1 | C42 | 300 | 22 | 441.1667 | 688.9846 | 0.880964 | 17.70546 | open-field | 6 | Paired |
| 2021 | 1 | C42 | 300 | 15 | 466.6111 | 1507.717 | 2.701867 | 35.52492 | mirror | 6 | Paired |
| 2021 | 2 | C42 | 300 | 61 | 800.6667 | 3525.371 | 3.793076 | 54.05191 | open-field | 6 | Paired |
| 2021 | 2 | C42 | 300 | 21 | 504.2778 | 2052.11 | 3.186035 | 37.98337 | mirror | 6 | Paired |
| 2021 | 3 | C42 | 300 | 24 | 542.6667 | 2321.543 | 3.946323 | 49.21927 | mirror | 6 | Paired |
| 2021 | 1 | C46 | 300 | 56 | 673.3333 | 2964.961 | 3.66478 | 43.94704 | open-field | 4.54 | Paired |
| 2021 | 2 | C46 | 295 | 51 | 696.7222 | 2879.566 | 3.68769 | 47.60838 | open-field | 4.54 | Paired |
| 2021 | 1 | C46 | 160 | 3 | 363.6667 | 679.7442 | 1.870033 | 30.16558 | mirror | 4.54 | Paired |
| 2021 | 2 | C46 | 300 | 15 | 511.3889 | 2113.91 | 3.420512 | 42.73746 | mirror | 4.54 | Paired |
| 2021 | 3 | C46 | 195 | 15 | 535.8889 | 2414.221 | 3.87511 | 58.40187 | mirror | 4.54 | Paired |
| 2020 | 1 | C10 | 300 | 148 | 750.9444 | 4808.95 | 5.015809 | 100.5268 | open-field | 4.91 | Paired |
| 2020 | 2 | C10 | 300 | 105 | 731.2778 | 6081.116 | 7.596948 | 164.5118 | open-field | 4.91 | Paired |
| 2020 | 3 | C10 | 300 | 87 | 696.1111 | 4667.404 | 5.92903 | 131.2628 | open-field | 4.91 | Paired |
| 2020 | 1 | C11 | 300 | 80 | 713.6111 | 4648.499 | 6.139779 | 113.8344 | open-field | 6.39 | Paired |
| 2020 | 2 | C11 | 300 | 83 | 703.2222 | 4312.373 | 6.04671 | 116.66 | open-field | 6.39 | Paired |
| 2020 | 3 | C11 | 47 | 103 | 754.3333 | 5588.784 | 7.17312 | 111.0023 | open-field | 6.39 | Paired |
| 2020 | 1 | C13 | 300 | 88 | 857.3889 | 3987.692 | 4.906633 | 115.57 | open-field | 8.23 | Paired |
| 2020 | 2 | C13 | 176 | 12 | 915 | 4537.416 | 3.568492 | 92.25479 | open-field | 8.23 | Paired |
| 2020 | 3 | C13 | 300 | 28 | 935.1667 | 4351.281 | 4.923587 | 139.4121 | open-field | 8.23 | Paired |
| 2020 | 1 | C14 | 188 | 69 | 645.6667 | 3937.549 | 5.266245 | 92.93332 | open-field | 5.99 | Paired |
| 2020 | 2 | C14 | 119 | 81 | 723.0556 | 3893.975 | 4.682057 | 85.42004 | open-field | 5.99 | Paired |
| 2020 | 1 | C15 | 300 | 81 | 673.3889 | 3599.253 | 4.730128 | 97.67803 | open-field | 6.43 | Paired |
| 2020 | 2 | C15 | 300 | 63 | 721.1111 | 4969.376 | 6.693749 | 126.3209 | open-field | 6.43 | Paired |
| 2020 | 3 | C15 | 300 | 92 | 665 | 5303.893 | 6.473877 | 160.5027 | open-field | 6.43 | Paired |
| 2020 | 1 | C16 | 135 | 20 | 615.2222 | 3674.935 | 7.973777 | 177.7617 | open-field | 8.17 | Paired |
| 2020 | 2 | C16 | 300 | 56 | 652.5556 | 3634.731 | 6.795475 | 145.3697 | open-field | 8.17 | Paired |
| 2020 | 3 | C16 | 300 | 87 | 761 | 5298.336 | 7.493624 | 157.2116 | open-field | 8.17 | Paired |
| 2020 | 1 | C17 | 300 | 68 | 644.0556 | 3959.282 | 5.485753 | 181.4423 | open-field | 5.14 | Paired |
| 2020 | 2 | C17 | 300 | 108 | 696.2222 | 5006.971 | 6.163651 | 87.74306 | open-field | 5.14 | Paired |
| 2020 | 1 | C2 | 272 | 76 | 706.4444 | 3734.143 | 4.8284 | 97.4398 | open-field | 6.4 | Paired |
| 2020 | 2 | C2 | 67 | 99 | 766.1111 | 4085.823 | 5.789511 | 125.9642 | open-field | 6.4 | Paired |
| 2020 | 3 | C2 | 300 | 101 | 722.7778 | 4118.963 | 4.188123 | 82.41101 | open-field | 6.4 | Paired |
| 2020 | 1 | C3 | 300 | 50 | 716 | 3313.397 | 2.04891 | 56.44278 | open-field | 5.56 | Paired |
| 2020 | 2 | C3 | 300 | 66 | 649.8889 | 2747.241 | 3.980682 | 89.87963 | open-field | 5.56 | Paired |
| 2020 | 3 | C3 | 286 | 53 | 647.0556 | 3203.369 | 5.443285 | 118.0852 | open-field | 5.56 | Paired |
| 2020 | 1 | C4 | 300 | 104 | 726.3333 | 4024.238 | 5.089794 | 99.81453 | open-field | 6.37 | Paired |
| 2020 | 2 | C4 | 280 | 100 | 730.0556 | 3813.947 | 8.20365 | 148.3038 | open-field | 6.37 | Paired |
| 2020 | 3 | C4 | 300 | 102 | 771.1111 | 5050.602 | 6.831162 | 126.9151 | open-field | 6.37 | Paired |
| 2020 | 1 | C8 | 300 | 67 | 706.3333 | 3631.807 | 4.900938 | 127.8923 | open-field | 10.41 | Paired |
| 2020 | 2 | C8 | 300 | 82 | 781.1667 | 6769.897 | 8.330472 | 226.3465 | open-field | 10.41 | Paired |
| 2020 | 3 | C8 | 300 | 37 | 709.7778 | 5316.314 | 7.463958 | 210.3363 | open-field | 10.41 | Paired |
| 2020 | 1 | C9 | 300 | 82 | 754.5556 | 5070.668 | 6.226426 | 147.8791 | open-field | 10.65 | Paired |
| 2020 | 2 | C9 | 300 | 122 | 765.6667 | 6405.362 | 8.523729 | 205.3882 | open-field | 10.65 | Paired |
| 2020 | 3 | C9 | 300 | 100 | 835.7778 | 7824.342 | 8.974832 | 240.0346 | open-field | 10.65 | Paired |
| 2020 | 1 | S1 | 234 | 94 | 713.8889 | 9739.169 | 15.13852 | 421.061 | open-field | 6.28 | Paired |
| 2020 | 2 | S1 | 110 | 82 | 672.8333 | 6350.864 | 10.09095 | 158.0746 | open-field | 6.28 | Paired |
| 2020 | 3 | S1 | 11 | 39 | 629.7222 | 3337.567 | 6.249771 | 85.93279 | open-field | 6.28 | Paired |
| 2020 | 1 | S10 | 300 | 62 | 846.5 | 4635.969 | 5.03594 | 121.2492 | open-field | 6.86 | Paired |
| 2020 | 2 | S10 | 300 | 77 | 919.4444 | 5352.323 | 5.276269 | 73.48477 | open-field | 6.86 | Paired |
| 2020 | 3 | S10 | 11 | 81 | 778.4444 | 4310.348 | 4.927921 | 63.88709 | open-field | 6.86 | Paired |
| 2020 | 1 | S11 | 21 | 67 | 682.3889 | 3682.226 | 5.282617 | 107.802 | open-field | 7.14 | Paired |
| 2020 | 2 | S11 | 21 | 37 | 593.8333 | 2525.836 | 3.619957 | 51.51268 | open-field | 7.14 | Paired |
| 2020 | 1 | S17 | 300 | 53 | 645.7222 | 3018.485 | 4.04428 | 86.34936 | open-field | 8.95 | Paired |
| 2020 | 2 | S17 | 300 | 63 | 660.8333 | 3038.213 | 3.835115 | 55.24908 | open-field | 8.95 | Paired |
| 2020 | 3 | S17 | 300 | 57 | 644.4444 | 3105.916 | 5.262068 | 61.0527 | open-field | 8.95 | Paired |
| 2020 | 1 | S2 | 149 | 21 | 661 | 4808.828 | 7.046886 | 191.9619 | open-field | 6.27 | Paired |
| 2020 | 2 | S2 | 300 | 30 | 626.4444 | 3160.33 | 4.567671 | 100.8224 | open-field | 6.27 | Paired |
| 2020 | 3 | S2 | 14 | 11 | 537.6667 | 2037.517 | 5.164962 | 98.80643 | open-field | 6.27 | Paired |
| 2020 | 1 | S3 | 59 | 41 | 655.1111 | 4921.266 | 7.501485 | 171.2221 | open-field | 7.91 | Paired |
| 2020 | 2 | S3 | 300 | 89 | 688.5 | 5603.93 | 6.609305 | 102.0041 | open-field | 7.91 | Paired |
| 2020 | 3 | S3 | 240 | 22 | 609.1111 | 3600.1 | 4.10636 | 57.8893 | open-field | 7.91 | Paired |
| 2020 | 1 | S4 | 201 | 106 | 703.1111 | 5205.786 | 6.835106 | 138.372 | open-field | 6.72 | Paired |
| 2020 | 2 | S4 | 300 | 81 | 684.1667 | 5050.903 | 6.080191 | 104.9999 | open-field | 6.72 | Paired |
| 2020 | 3 | S4 | 213 | 41 | 621.3333 | 3997.488 | 4.865788 | 69.66964 | open-field | 6.72 | Paired |
| 2020 | 1 | S5 | 300 | 43 | 672.1667 | 8447.3 | 10.58591 | 285.2647 | open-field | 7.59 | Paired |
| 2020 | 2 | S5 | 300 | 64 | 678.9444 | 3687.572 | 4.158603 | 76.92041 | open-field | 7.59 | Paired |
| 2020 | 3 | S5 | 21 | 66 | 662.0556 | 5688.169 | 14.34725 | 239.5251 | open-field | 7.59 | Paired |
| 2020 | 1 | S6 | 7 | 6 | 593 | 2861.583 | 3.782438 | 81.21042 | open-field | 7.34 | Paired |
| 2020 | 2 | S6 | 15 | 105 | 810.9444 | 4457.807 | 4.673744 | 62.11535 | open-field | 7.34 | Paired |
| 2020 | 3 | S7 | 142 | 11 | 634.2778 | 5209.227 | 8.497091 | 236.9877 | open-field | 6.82 | Paired |
| 2020 | 1 | S7 | 264 | 4 | 627.5 | 3646.207 | 3.146374 | 63.36485 | open-field | 6.82 | Paired |
| 2020 | 2 | S7 | 300 | 91 | 754.6667 | 4519.341 | 5.960584 | 84.48011 | open-field | 6.82 | Paired |
| 2020 | 3 | S8 | 6 | 178 | 691 | 10304.01 | 11.27723 | 183.8021 | open-field | 7.94 | Paired |
| 2020 | 1 | S8 | 17 | 210 | 741.9444 | 12607.42 | 12.25105 | 163.4986 | open-field | 7.94 | Paired |
| 2020 | 2 | S8 | 8 | 234 | 810.6111 | 14935.74 | 13.33406 | 171.7372 | open-field | 7.94 | Paired |
| 2020 | 1 | S9 | 133 | 13 | 0 | 0 | 0 | 0 | open-field | 7.01 | Paired |
| 2020 | 2 | S9 | 300 | 21 | 0 | 0 | 0 | 0 | open-field | 7.01 | Paired |
| 2020 | 3 | S9 | 300 | 20 | 581.9444 | 4600.609 | 8.830707 | 274.8986 | open-field | 7.01 | Paired |
| 2020 | 1 | C10 | 300 | 26 | 512.9444 | 2056.616 | 3.669451 | 57.85787 | mirror | 4.91 | Paired |
| 2020 | 2 | C10 | 300 | 22 | 554.7222 | 1935.925 | 3.383826 | 66.78976 | mirror | 4.91 | Paired |
| 2020 | 3 | C10 | 300 | 31 | 598.8889 | 2989.024 | 4.942614 | 92.87726 | mirror | 4.91 | Paired |
| 2020 | 1 | C11 | 130 | 15 | 607.5 | 3091.141 | 5.13129 | 83.88575 | mirror | 6.39 | Paired |
| 2020 | 2 | C11 | 300 | 34 | 636.3333 | 4358.003 | 7.593074 | 94.96092 | mirror | 6.39 | Paired |
| 2020 | 3 | C11 | 195 | 28 | 624.2778 | 4434.087 | 7.067677 | 137.2243 | mirror | 6.39 | Paired |
| 2020 | 1 | C13 | 300 | 12 | 559.1667 | 2393.806 | 3.867032 | 81.86449 | mirror | 8.23 | Paired |
| 2020 | 2 | C13 | 268 | 7 | 567.7778 | 2046.234 | 3.768354 | 66.35331 | mirror | 8.23 | Paired |
| 2020 | 3 | C13 | 0 | 7 | 579.5 | 2779.101 | 4.502308 | 113.4171 | mirror | 8.23 | Paired |
| 2020 | 1 | C14 | 10 | 24 | 557.6667 | 2879.333 | 4.434282 | 93.50223 | mirror | 5.99 | Paired |
| 2020 | 2 | C14 | 300 | 10 | 605.0556 | 2422.56 | 3.83309 | 67.16389 | mirror | 5.99 | Paired |
| 2020 | 3 | C14 | 0 | 38 | 588.7778 | 5162.024 | 8.562453 | 207.4645 | mirror | 5.99 | Paired |
| 2020 | 1 | C15 | 65 | 14 | 539.3333 | 1713.753 | 2.500851 | 48.15987 | mirror | 6.43 | Paired |
| 2020 | 2 | C15 | 224 | 24 | 582.7778 | 2717.686 | 4.169329 | 67.96759 | mirror | 6.43 | Paired |
| 2020 | 3 | C15 | 75 | 46 | 640.5556 | 4720.134 | 6.79838 | 146.6902 | mirror | 6.43 | Paired |
| 2020 | 1 | C16 | 300 | 33 | 507.1667 | 2823.1 | 5.229583 | 74.39221 | mirror | 8.17 | Paired |
| 2020 | 2 | C16 | 300 | 59 | 677.6667 | 3972.815 | 5.782104 | 81.06639 | mirror | 8.17 | Paired |
| 2020 | 3 | C16 | 14 | 50 | 638 | 6499.725 | 10.0052 | 243.915 | mirror | 8.17 | Paired |
| 2020 | 1 | C17 | 300 | 14 | 583 | 2437.136 | 3.773272 | 69.78 | mirror | 5.14 | Paired |
| 2020 | 2 | C17 | 300 | 9 | 563.5 | 2465.415 | 5.346072 | 140.0117 | mirror | 5.14 | Paired |
| 2020 | 3 | C17 | 300 | 45 | 616.8333 | 3634.103 | 5.733528 | 122.5603 | mirror | 5.14 | Paired |
| 2020 | 1 | C2 | 300 | 10 | 587.5556 | 2848.234 | 4.753904 | 85.69876 | mirror | 6.4 | Paired |
| 2020 | 2 | C2 | 300 | 1 | 584.9444 | 2392.796 | 1.828889 | 39.09674 | mirror | 6.4 | Paired |
| 2020 | 3 | C2 | 17 | 41 | 614.1111 | 4657.644 | 6.996322 | 150.9202 | mirror | 6.4 | Paired |
| 2020 | 1 | C3 | 300 | 7 | 431.6667 | 1048.638 | 1.780715 | 37.65914 | mirror | 5.56 | Paired |
| 2020 | 2 | C3 | 300 | 41 | 605.5556 | 3109.939 | 4.895567 | 70.93285 | mirror | 5.56 | Paired |
| 2020 | 3 | C3 | 300 | 23 | 554.7778 | 2233.889 | 3.350814 | 91.90521 | mirror | 5.56 | Paired |
| 2020 | 1 | C4 | 142 | 38 | 628 | 4533.872 | 6.831207 | 96.31872 | mirror | 6.37 | Paired |
| 2020 | 2 | C4 | 201 | 22 | 637.1111 | 3661.736 | 6.459523 | 79.77186 | mirror | 6.37 | Paired |
| 2020 | 3 | C4 | 39 | 26 | 595.1111 | 4382.869 | 6.786909 | 164.5075 | mirror | 6.37 | Paired |
| 2020 | 1 | C8 | 300 | 9 | 554.1111 | 1929.928 | 2.704818 | 63.79412 | mirror | 10.41 | Paired |
| 2020 | 2 | C8 | 300 | 3 | 575.1667 | 2552.532 | 3.820945 | 84.29674 | mirror | 10.41 | Paired |
| 2020 | 3 | C8 | 300 | 10 | 622.6667 | 5911.199 | 8.122524 | 216.5948 | mirror | 10.41 | Paired |
| 2020 | 1 | C9 | 48 | 27 | 501.1667 | 2566.981 | 4.847495 | 74.81069 | mirror | 10.65 | Paired |
| 2020 | 2 | C9 | 285 | 59 | 662.1111 | 3957.832 | 5.785684 | 77.06699 | mirror | 10.65 | Paired |
| 2020 | 3 | C9 | 300 | 21 | 589.3889 | 3750.267 | 7.229426 | 175.781 | mirror | 10.65 | Paired |
| 2020 | 1 | S1 | 151 | 35 | 626 | 5696.503 | 8.097643 | 136.9176 | mirror | 6.28 | Paired |
| 2020 | 2 | S1 | 300 | 77 | 719.4444 | 5164.525 | 7.042788 | 81.13177 | mirror | 6.28 | Paired |
| 2020 | 3 | S1 | 8 | 8 | 624.2778 | 6391.725 | 14.98763 | 341.1662 | mirror | 6.28 | Paired |
| 2020 | 1 | S10 | 300 | 3 | 560.3333 | 2909.638 | 2.819087 | 51.01726 | mirror | 6.86 | Paired |
| 2020 | 2 | S10 | 300 | 12 | 559.6111 | 2721.649 | 4.251068 | 81.05638 | mirror | 6.86 | Paired |
| 2020 | 3 | S10 | 300 | 5 | 590.9444 | 2891.613 | 8.225239 | 129.6585 | mirror | 6.86 | Paired |
| 2020 | 1 | S11 | 190 | 6 | 595.1111 | 2924.371 | 4.953569 | 72.41923 | mirror | 7.14 | Paired |
| 2020 | 2 | S11 | 205 | 2 | 589.6667 | 3792.547 | 7.824611 | 141.9464 | mirror | 7.14 | Paired |
| 2020 | 3 | S11 | 7 | 2 | 582.8333 | 2697.641 | 18.6393 | 360.1809 | mirror | 7.14 | Paired |
| 2020 | 1 | S12 | 96 | 71 | 716.6111 | 3244.196 | 4.376998 | 54.36721 | mirror | 7.73 | Paired |
| 2020 | 2 | S12 | 0 | 35 | 644.5556 | 2279.511 | 3.167057 | 45.98298 | mirror | 7.73 | Paired |
| 2020 | 1 | S14 | 300 | 26 | 613.7778 | 2934.629 | 4.635847 | 58.14055 | mirror | 7.74 | Paired |
| 2020 | 2 | S14 | 300 | 7 | 563.7222 | 2193.465 | 4.257513 | 86.67862 | mirror | 7.74 | Paired |
| 2020 | 1 | S17 | 18 | 14 | 584.7222 | 2716.896 | 3.489976 | 64.42118 | mirror | 8.95 | Paired |
| 2020 | 2 | S17 | 300 | 4 | 538.0556 | 2442.192 | 3.943619 | 98.0624 | mirror | 8.95 | Paired |
| 2020 | 3 | S17 | 300 | 41 | 643.4444 | 2984.599 | 4.268559 | 65.57544 | mirror | 8.95 | Paired |
| 2020 | 1 | S2 | 300 | 5 | 527.4444 | 2317.845 | 3.491242 | 54.41876 | mirror | 6.27 | Paired |
| 2020 | 2 | S2 | 300 | 13 | 615.1667 | 4499.1 | 5.790859 | 110.8527 | mirror | 6.27 | Paired |
| 2020 | 3 | S2 | 300 | 3 | 597.6111 | 3869.231 | 11.40687 | 314.8404 | mirror | 6.27 | Paired |
| 2020 | 1 | S3 | 108 | 2 | 583.6667 | 3030.307 | 6.824477 | 81.75776 | mirror | 7.91 | Paired |
| 2020 | 2 | S3 | 300 | 1 | 569.5 | 2675.527 | 2.124559 | 41.74754 | mirror | 7.91 | Paired |
| 2020 | 3 | S3 | 147 | 46 | 636.7778 | 8996.053 | 14.41467 | 425.3646 | mirror | 7.91 | Paired |
| 2020 | 1 | S4 | 262 | 7 | 593.6667 | 3004.331 | 5.690275 | 85.27627 | mirror | 6.72 | Paired |
| 2020 | 2 | S4 | 291 | 1 | 581.3889 | 2903.791 | 2.305816 | 40.82998 | mirror | 6.72 | Paired |
| 2020 | 3 | S4 | 51 | 13 | 608.6667 | 5929.903 | 11.92697 | 313.4928 | mirror | 6.72 | Paired |
| 2020 | 1 | S5 | 8 | 50 | 635.2222 | 5793.497 | 8.101346 | 126.9444 | mirror | 7.59 | Paired |
| 2020 | 2 | S5 | 300 | 84 | 726.5556 | 5239.752 | 7.105285 | 84.16554 | mirror | 7.59 | Paired |
| 2020 | 3 | S5 | 173 | 6 | 619.0556 | 5693.815 | 12.83519 | 225.5195 | mirror | 7.59 | Paired |
| 2020 | 1 | S6 | 300 | 23 | 607.2222 | 2729.81 | 4.401059 | 64.20256 | mirror | 7.34 | Paired |
| 2020 | 2 | S6 | 12 | 1 | 601.8333 | 3446.418 | 2.669572 | 49.03441 | mirror | 7.34 | Paired |
| 2020 | 3 | S6 | 7 | 4 | 551.0556 | 2128.327 | 6.84618 | 129.5746 | mirror | 7.34 | Paired |
| 2020 | 1 | S7 | 300 | 17 | 541.2778 | 2917.329 | 4.144517 | 75.49043 | mirror | 6.82 | Paired |
| 2020 | 2 | S7 | 300 | 6 | 582.0556 | 3819.492 | 4.990273 | 103.2824 | mirror | 6.82 | Paired |
| 2020 | 3 | S7 | 17 | 1 | 570.1667 | 3414.303 | 2.715271 | 70.48177 | mirror | 6.82 | Paired |
| 2020 | 1 | S8 | 166 | 24 | 627.7778 | 6702.421 | 18.3916 | 381.6804 | mirror | 7.94 | Paired |
| 2020 | 2 | S8 | 300 | 77 | 697.4444 | 5659.19 | 7.892191 | 122.3499 | mirror | 7.94 | Paired |
| 2020 | 3 | S8 | 15 | 96 | 692.3889 | 7470.106 | 10.73249 | 164.0169 | mirror | 7.94 | Paired |
| 2020 | 1 | S9 | 300 | 1 | 0 | 0 | 0 | 0 | mirror | 7.01 | Paired |
| 2020 | 2 | S9 | 14 | 1 | 0 | 0 | 0 | 0 | mirror | 7.01 | Paired |
| 2020 | 3 | S9 | 22 | 3 | 0 | 0 | 0 | 0 | mirror | 7.01 | Paired |
